# The Acceptance/Avoidance-Promoting Experiences Questionnaire (APEQ): A theory-based approach to psychedelic drugs’ effects on psychological flexibility

**DOI:** 10.1177/02698811211073758

**Published:** 2022-03-07

**Authors:** Max Wolff, Lea J Mertens, Marie Walter, Sören Enge, Ricarda Evens

**Affiliations:** 1 MIND Foundation, Berlin, Germany; 2 Charité – Universitätsmedizin Berlin, corporate member of Freie Universität Berlin and Humboldt-Universität zu Berlin, Department of Psychiatry and Psychotherapy, Campus Charité Mitte, Berlin, Germany; 3 Faculty of Psychology, Dresden University of Technology, Dresden, Germany; 4 Department of Molecular Neuroimaging, Central Institute of Mental Health, Medical Faculty Mannheim, University of Heidelberg, Mannheim, Germany; 5 Department of Psychology, University of Mainz, Mainz, Germany; 6 Department of Psychology, MSB Medical School Berlin, Berlin, Germany

**Keywords:** Avoidance, acceptance, LSD, psilocybin, ayahuasca

## Abstract

**Background:**

Many benefits and some harms associated with psychedelic use could be attributable to these drugs’ acceptance/avoidance-promoting effects and corresponding changes in psychological flexibility. Underlying psychological mechanisms are insufficiently understood.

**Aim:**

The purpose of this study was the validation of a psychological model of acceptance/avoidance-promoting psychedelic experiences, which included the development of a theory-based self-report instrument: the Acceptance/Avoidance-Promoting Experiences Questionnaire (APEQ). Its two main scales, acceptance-related experience (ACE) and avoidance-related experience (AVE), represent the theorized model’s core constructs. We aimed to test the model’s central assumptions of complementarity (ACE and AVE may occur alternatingly but not simultaneously, and are therefore empirically independent), intertwinedness (subaspects within ACE and AVE are mutually contingent and therefore highly inter-correlated), context-dependence (ACE and AVE depend on context factors) and interaction (longer-term outcomes depend on the interplay between ACE and AVE).

**Method:**

A bilingual retrospective online survey including 997 English- and 836 German-speaking participants. Each participant reported on one psychedelic experience occasioned by lysergic acid diethylamide (LSD), psilocybin, mescaline, or ayahuasca.

**Results:**

Whereas ACE and AVE were found to be relatively independent aspects of participants’ reported psychedelic experiences (complementarity), their subaspects were mostly distinguishable but strongly correlated among each other (intertwinedness). Therapeutic, escapist, and hedonic use motives were differentially associated with ACE and AVE (context-dependence), which were in turn associated with retrospective changes in psychological flexibility following participants’ reported experiences. The positive association between ACE and increased psychological flexibility was significantly moderated by AVE (interaction).

**Conclusion:**

These results provide an initial validation of the APEQ and its underlying theoretical model, suggesting the two can help clarify the psychological mechanisms of psychedelic-induced benefits and harms. Both should be further investigated in prospective-longitudinal and clinical studies.

## Introduction

Classic serotonergic psychedelics such as psilocybin, lysergic acid diethylamide (LSD), and dimethyltryptamine (DMT; the principal pharmacological agent in ayahuasca) are currently being investigated as promising treatments for a wide range of mental health conditions including major depression (Carhart-Harris et al., 2016, 2018a, 2021; Davis et al., 2020b; Palhano-Fontes et al., 2019), psychological distress associated with chronic or terminal illness (Agin-Liebes et al., 2020; Anderson et al., 2020; Gasser et al., 2014; Griffiths et al., 2016; Grob et al., 2011; Ross et al., 2016), obsessive-compulsive disorder (Moreno et al., 2006), and substance use disorders (Bogenschutz et al., 2015; Johnson et al., 2014, 2017). Converging evidence from psychometric tests (Carhart-Harris et al., 2021; Griffiths et al., 2016), neuroimaging studies (Mertens et al., 2020; Roseman et al., 2018), and qualitative interviews with patients (Belser et al., 2017; Gasser et al., 2015; Watts et al., 2017) suggests that these drugs’ therapeutic effects are associated with shifts from excessive *experiential avoidance* (the tendency to avoid or control aversive emotions, sensations, thoughts, or memories despite negative consequences) toward more *acceptance* (the converse ability to allow, tolerate, and engage with these experiences). Acceptance is fundamental to psychological flexibility, a crucial aspect of emotional well-being and a central target of psychotherapy (most notably the “third wave” cognitive-behavioral therapies; Kashdan and Rottenberg, 2010). Observational and experimental studies in healthy individuals (Smigielski et al., 2019; Soler et al., 2016, 2018) as well as online surveys (Davis et al., 2020a, 2021; Zeifman et al., 2020) suggest that the acceptance-promoting effects of psychedelics are not restricted to their clinical uses.

Much less discussed in the literature, presumably because severe psychedelic-induced harm is relatively rare (Nutt et al., 2010; Van Amsterdam et al., 2015), is the possibility that experiential avoidance is not reduced but rather enduringly increased following a psychedelic experience, leading to psychological inflexibility, impaired well-being, or even mental illness. However, this scenario seems quite plausible given psychedelic drugs’ highly context-dependent effects (Carhart-Harris et al., 2018b), and could explain some of the psychological harms that are indeed sometimes occasioned by psychedelic use in recreational, ceremonial, or underground therapy settings (Cohen, 1966; Strassman, 1984).

In the following, we present a unified theoretical model that aims to specify the complementary psychological processes underlying both the acceptance- and the avoidance-promoting effects that can be induced by psychedelic experiences. We then describe the development of a culturally decentered self-report measure for quantifying these processes, the Acceptance/Avoidance-Promoting Experiences Questionnaire (APEQ), and report results from a bilingual online survey that was conducted with the purpose of validating the APEQ and testing the central assumptions of its theoretical basis.

### A unified psychological model of acceptance- and avoidance-promoting psychedelic experiences

**Figure 1.**
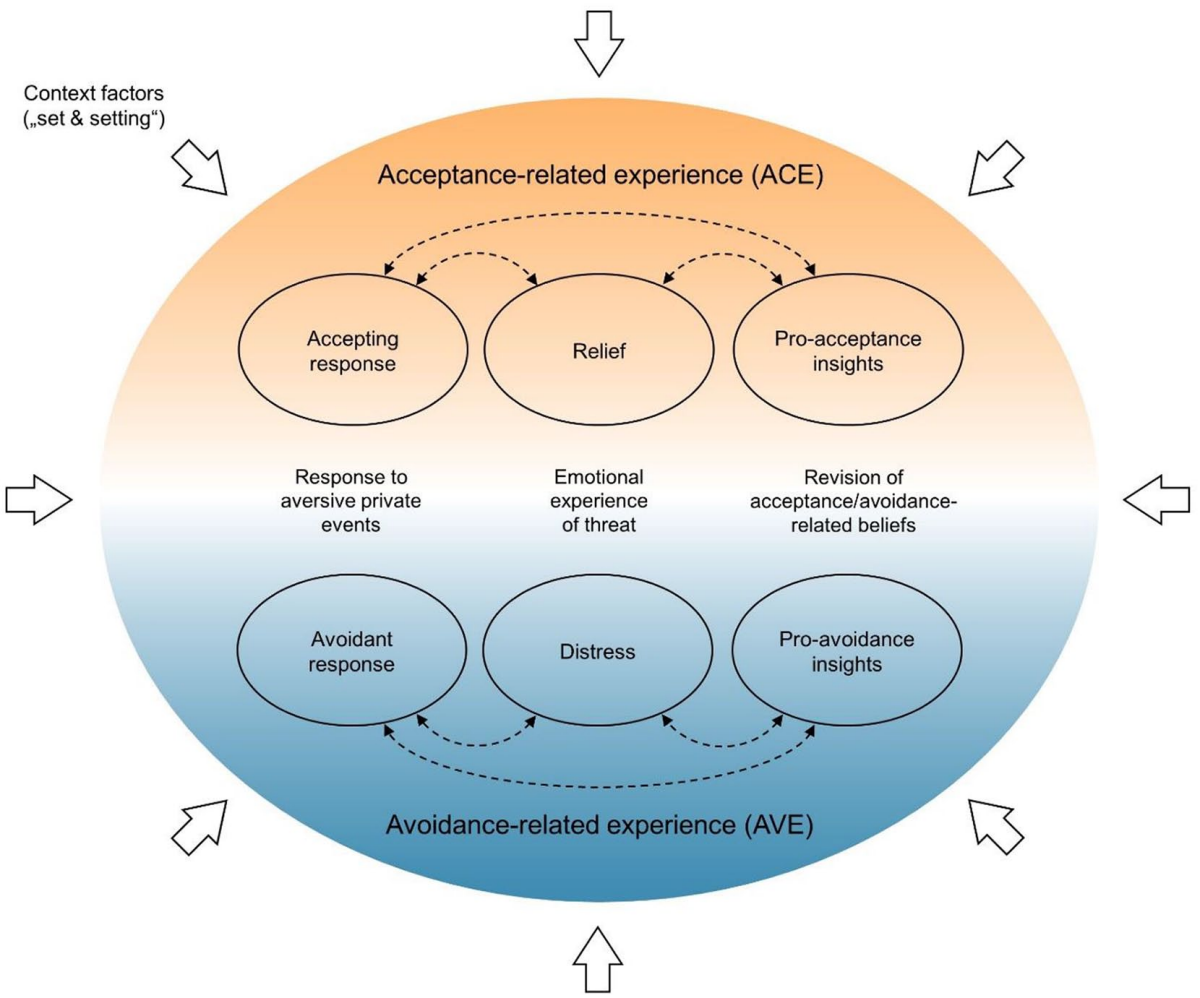
Schematic illustration of the proposed psychological model of acceptance- and avoidance-promoting psychedelic experiences. Intertwined subaspects of acceptance-related experience (ACE) and avoidance-related experience (AVE) are illustrated as ellipses connected by dashed arrows. Vertically neighboring ellipses represent the pairs of complementary subaspects. It is assumed that complementary sub aspects cannot occur simultaneously but may alternate over the course of a given experience.

Figure 1 illustrates the proposed conceptual model. Based on a previous theoretical argument that was more exclusively focused on acceptance-promoting effects (Wolff et al., 2020), the present model has been extended and applies to avoidance-promoting psychedelic experiences as well. In a general sense, the model assumes that longer-term changes in acceptance and/or avoidance (as well as related constructs such as psychological flexibility or mindfulness) following psychedelic states result from a highly context-dependent learning process that can involve aspects of both *acceptance-related experience* (ACE) and *avoidance-related experience* (AVE). ACE and AVE include pairs of complementary subaspects within three distinguishable domains which are outlined in the following: (1) response to aversive private events, (2) emotional experience of threat, and (3) revision of acceptance/avoidance-related beliefs.

#### Response to aversive private events

At any moment over the course of a given psychedelic experience, it may occur that the individual is confronted with an aversive *private event* (i.e. an emotion, sensation, perception, thought, or memory). In that case, the individual can show either an avoidant response (e.g. trying to suppress a painful memory) or an accepting response (allowing the memory to surface and letting associated emotions unfold freely). This choice is thought to depend, in part, on the individual’s learning history and related personality or temperamental traits, but also substantially on more proximal context factors such as acceptance-related intentions, trainable dispositions for accepting responses (e.g. mindfulness capabilities or the skill of “leaning into” aversive experiences), the presence of a trusted person who can provide respective cues (e.g. a guide or therapist), and the appraisal of the immediate situation as safe rather than threatening. At a closer look, most recommendations according to current standard protocols for psychedelic therapy (Garcia-Romeu and Richards, 2018), guidelines for human research with psychedelics (Johnson et al., 2008), and psychedelic harm reduction practices (Gorman et al., 2021; Oak et al., 2015) can be understood as measures for establishing a context that favors accepting over avoidant responses to aversive private events that may arise in the psychedelic state.

#### Emotional experience of threat

An additional potential influence on the disposition for accepting versus avoidant responses are emotional experiences related to perceived threat (distress) and its resolution (relief). Theories of avoidance commonly assume that avoidance of threatening stimuli is learned by operant conditioning, as avoidant responses typically result in the omission of aversive events that would otherwise occur (i.e. negative reinforcement; Maia, 2010; Mowrer, 1951). In psychedelic states, however, this rule can apparently be suspended or even reversed: Rather than bringing relief from threatening private events, avoidant responses to aversive psychedelic experiences often lead to increased distress, presumably especially in uncertain stimulus environments (Wolff et al., 2020). While the precise mechanisms underlying this “avoidance sensitivity” are not well understood and remain to be studied experimentally, we have proposed that the effect emerges when psychedelic-induced belief relaxation (Carhart-Harris and Friston, 2019) allows avoidance-related motivational processes (e.g. implicit representations of avoidance goals) to infiltrate and distort ongoing perceptual belief updating in such a way that the phenomenal experience becomes dominated by threat-related memory contents (Wolff et al., 2020). Thereby, for instance, the attempt to suppress an arising emotion may induce unsettling bodily sensations, sinister imagery, and so on. Conversely, shifting from avoidant responding towards more acceptance can lead to immediate relief from such distress. Under favorable conditions, these reward contingencies can conceivably impel a shaping-like^1^ operant process of “learning to let go” where negative reinforcement of accepting responses leads up to a conditioned state of emotional openness that is largely free from avoidant reactions (Wolff et al., 2020). This can be accompanied by intense feelings of relief, release, or liberation, and may entail a sense of *emotional breakthrough* (Roseman et al., 2019).

However, the described process presumably depends on the aforementioned context factors (e.g. intentions, capabilities, and presence of a trusted person). Under less favorable conditions where “learning to let go” is impeded, persistent avoidance may instead contribute to the escalation and maintenance of distressful states that can be referred to as *challenging experiences* (Barrett et al., 2016; Carbonaro et al., 2016), and are characterized by panic, desperation, and psychological struggle. In our conceptualization, one critical factor deciding between these two complementary states is whether one’s affective experience of threat is attributed to the arising private events themselves or rather to one’s own way of responding to them. In the distressful state, the individual attributes the situation’s aversiveness exclusively to certain private events that are arising unwantedly and uncontrollably (external attribution). Parallels between this condition and “learned helplessness” (Seligman, 1972), that is, perceived lack of control over reinforcement, may suggest that depressed or demoralized individuals will remain in this state for longer periods of time (and point toward another potential therapeutic mechanism). Approaching “breakthrough”, the individual begins to acquire some sense of the role of their own avoidant responses in determining the affective outcome (internal attribution), which may then increase spontaneous accepting responses and thereby enable the described operant process.

#### Revision of acceptance/avoidance-related beliefs

Current theories conceptualize the psychedelic experience as a “pivotal mental state,” that is, a temporary window of heightened neural and psychological plasticity where deeply held beliefs are “relaxed” (destabilized and sensitive to new information), and thus become amenable to enduring change (Brouwer and Carhart-Harris, 2020; Carhart-Harris and Friston, 2019). The direction and magnitude of such belief revision are assumed to depend on the quality of the psychedelic experience, particularly on its affective tone and relatedness to the respective beliefs (Brouwer and Carhart-Harris, 2020). Accordingly, the above-described emotional experiences related to perceived threat from private events, distress and relief, should be considered essential determinants of how avoidance- and acceptance-related beliefs will be affected after a psychedelic experience. Enhanced psychological flexibility can thus be understood as a consequence of pivotal-state learning experiences where increasingly accepting responses to aversive private events are accompanied by an affective shift from distress to relief, leading to a de-weighting of pro-avoidance beliefs.

In addition, unique opportunities for revising avoidance-related belief structures likely arise when the described operant process of learning acceptance occurs in the presence of context factors that deploy psychedelics’ potential to elicit highly emotional, personally meaningful experiences (e.g. therapeutic intentions and music; Barrett et al., 2018; Garcia-Romeu and Richards, 2018; Hartogsohn, 2018; Swanson, 2018). Under such favorable circumstances, the conditioned ability to confront the arising emotional material in a largely non-avoidant manner may lead to acceptance-promoting insights similar to those obtained through the kinds of “corrective experiences” (Goldfried, 2012) that are purposefully induced in psychotherapy. Characteristic episodes of deep introspection where intense emotions are met with exceptional openness are commonly reported, and indeed often described in ways suggestive of underlying psychotherapeutic processes, by psychedelic therapy patients and others who have used psychedelics with therapeutic intent (Breeksema et al., 2020). That these episodes involve corrective experiences is in line with the view that the therapeutic potential of psychedelic states arises from the same general change mechanisms that are shared by all effective psychotherapies (Jungaberle et al., 2008; Nayak and Johnson, 2020). Following a distinction introduced by Grawe (1997) based on broad empirical psychotherapy research, corrective experiences of *mastery/coping* can strengthen the belief of being able to accommodate situations, feelings, thoughts, memories, etc. that were previously experienced as too painful or frightening to cope with. Through a second type of corrective experience, termed *clarification of meaning* (Grawe, 1997; Grosse Holtforth et al., 2006), the individual gains awareness and understanding of previously implicit fears and their associations with emotions and behavior. Increased awareness and understanding allow for questioning and revising the belief structures underlying these fears, which can lead to less negative appraisals of relevant situations, and thus, reduced avoidance. Patient accounts of dosing sessions suggest that mastery/coping and clarification experiences do in fact commonly occur within psychedelic states, at least in therapeutic settings (Belser et al., 2017; Breeksema et al., 2020; Gasser et al., 2015; Watts et al., 2017). Longer-term impacts of such corrective experiences are presumably amplified, or even made possible in the first place, by psychedelic-induced increases in plasticity. On the positive side, this implies opportunities for deep and enduring psychological change from excessive experiential avoidance toward more acceptance—even in otherwise rigid individuals whose avoidance-related beliefs are in normal waking consciousness resistant to corrective experiences (a common problem in conventional psychotherapies; Rief et al., 2015).

However, heightened plasticity may also aggravate potential harms to individuals who are exposed to adverse learning conditions while under the influence of psychedelics. Challenging experiences may then persist for extended periods of time or even the entire duration of the acute drug effect. Without the described operant process of learning acceptance, and without subsequent episodes of emotional openness, the individual may emerge from the pivotal state with enduringly strengthened (or newly induced) beliefs along the lines that certain private events are indeed too frightening or painful to cope with, that is, overextension (the opposite effect of a mastery/coping experience). Likewise, such experiences may bring not clarification of meaning but, to the contrary, obfuscation or confusion. As a result, the individual may acquire strengthened implicit fears and even entirely new “pathogenic meanings” (Frank and Frank, 1993), that is, previously unburdened topics become associated with severe threats to basic psychological needs (see Timmermann et al., 2021, for an illustrative example). In situations where these types of challenging experiences remain unresolved (i.e. overextension without a subsequent experience of mastery/coping or obfuscation/confusion without subsequent clarification), a temporary increase in the plasticity of avoidance-related belief systems would presumably have rather negative longer-term effects on the individual’s psychological flexibility.

### Overarching assumptions

Beyond the specific domains of response, emotional experience, and belief revision presented above, the outlined theoretical model has a set of overarching assumptions:

- *Complementarity*:^2^ ACE and AVE are conceptualized as complementary features of psychedelic experiences that cannot occur simultaneously (with respect to the same private event(s)) but may alternate over the course of a given experience. For example, one cannot simultaneously suppress and allow the surfacing of a certain memory, but doing both at different moments is possible. Because of their complementarity, ACE and AVE are assumed to be relatively independent empirically.
- *Intertwinedness*: In contrast, distinguishable subaspects within ACE and AVE are functionally intertwined, and therefore assumed to be strongly correlated. For instance, experiences of relief are probably both contingent on and conducive to accepting responses. Likewise, accepting responses and feelings of relief can lead to acceptance-promoting insights, which will in turn again increase the propensity for accepting responses. Because of these bidirectional effects, all three subaspects will often co-occur and correspond to one another in magnitude.
- *Context-dependence*: Given that acute responses to psychedelics are generally strongly influenced by context (“set and setting”; Carhart-Harris et al., 2018b; Hartogsohn, 2016), ACE and AVE are assumed to depend on context factors such as use motives and a therapeutic setting.
- *Interaction*: Longer-term changes in acceptance/avoidance and related outcomes (e.g. psychological flexibility) occasioned by psychedelic experiences are understood not (only) as additive effects of ACE and AVE, but also as the result of the interplay between the two. Most importantly, and relevant for clinical applications as well as harm reduction, even highly challenging experiences characterized by desperate resistance and intense distress may produce no longer-term increase in avoidance if a shift to acceptance (and thus relief) is eventually achieved.

### Introspection and interaction with the environment as ancillary factors

According to standard protocols for dosing sessions in current psychedelic therapy research, which are largely consistent with the historical “psychedelic model” (Osmond, 1957; Pahnke et al., 1970), patients are encouraged to direct attention toward the inner experience of emotions, thoughts and bodily sensations while lying on a bed or sofa wearing eyeshades and headphones (Garcia-Romeu and Richards, 2018). Interactions with the environment such as moving the body, observing the visual surroundings, and talking to the therapists are thus limited by default, but can be initiated by the patient at any moment, especially when handling psychological distress (e.g. seeking distraction, reassurance, or guidance). In a previous article, we have pointed out that switching from introspection to interaction in such settings can serve as an effective avoidance strategy for tuning down the intensity of aversive psychedelic experiences by means of active inference (acting on the environment to reduce sensory uncertainty) and well-defined sensory input (Wolff et al., 2020; for mechanisms, see Mediano et al., 2020; Pink-Hashkes et al., 2017). Therefore, to the degree that it is used as an avoidance strategy, interaction with the environment can be expected to show a differential pattern of associations with AVE subaspects. We hypothesize that, in contexts resembling psychedelic therapy, levels of interaction will be negatively correlated with distress but positively correlated with avoidant responses and pro-avoidance insights.

### The present study

The primary aim of this study was the initial empirical validation of the proposed conceptual model, that is, to confirm its components (distinguishable subaspects of ACE and AVE as well as the ancillary factors introspection and interaction) and verify the overarching assumptions of complementarity, intertwinedness, context-dependence, and interaction. To this end, we developed a new self-report questionnaire based on the model, the APEQ, and conducted a bilingual online survey where it was applied among measures of context (use intentions) and longer-term outcomes (changes in psychological flexibility). Since the APEQ is a theory-based research instrument, this study aims to serve its validation as well as the validation of the underlying theory.

A common problem with the traditional successive approach for questionnaire development (i.e. developing an instrument in one language and subsequent translation into other languages) is that the resulting culture-centeredness (e.g. idiosyncratic expressions that are hard to translate) limits the achievable equivalence of translated versions (Tanzer, 2005). To avoid this problem, here we employed a parallel development approach in English and German.

## Methods

### APEQ development

In accordance with the theorized conceptual model described above (see Figure 1), the APEQ was designed to include the two main scales *ACE* and *AVE*, each composed of three subscales corresponding to the respective complementary subaspects in the three domains (1) response to aversive private events (*accepting response* and *avoidant response*), (2) emotional experience of threat (*relief* and *distress*), and (3) revision of acceptance/avoidance-related beliefs (*pro-acceptance insights* and *pro-avoidance insights*). In addition to the main scales, which represent core constructs of the model, two ancillary scales *introspection* and *interaction*, corresponding to the homonymous ancillary factors, were developed. A visual analogue scale (0–100, with incremental units of one) with verbal anchors at 0 (“No, not at all”) and 100 (“Yes, extremely or absolutely”) was chosen as the response format.

#### Formulation of items

Based on the pre-defined theoretical constructs explained above, and following general recommendations for item formulation (Elson, 2017), the authors of the present article crafted a pool of 57 candidate items. Each item was specifically designed for one subscale or ancillary scale, yielding eight subpools comprising between five and nine items. An external expert (R Zeifman) assessed the item pool and provided feedback for refinements at an early stage of development.

To accommodate the assumption of complementarity, item development was guided by the requirement to allow for empirical independence between the complementary subscales. Hence, each item statement had to be formulated in such a way that endorsement of the item would not automatically imply non-endorsement of items in the respective complementary subscale. To achieve this, items were not allowed to include strong generalizations. For instance, “I was open to difficult sensations or emotional states” was allowed, whereas the more generalizing “I was *constantly* open to difficult sensations or emotional states” would have been insufficiently independent of (allowed) complementary items such as “I made efforts to avoid or control difficult feelings.” Likewise, negative items (e.g. “I was *not* open to difficult sensations or emotional states”) were not allowed due to their generalizing nature. To accommodate the large variety of private events that can be objects of acceptance and avoidance, item generation was aimed at including statements referring to a broad range of cognitive, emotional, mnemonic, and sensory experiences. Individual items were allowed to cover private events of multiple domains.

#### Simultaneous item development in English and German languages

To avoid the disadvantages associated with item translation and ensure maximum equivalence between the English and German APEQ versions, all items were crafted, discussed, and revised simultaneously in both languages. Following recommendations for simultaneous test development (Tanzer, 2005), the item pool underwent two rounds of review by a bilingual task force drafted from staff members of the MIND Foundation. The task force was composed of three native English speakers, three native German speakers (each proficient in the respective other language), and one native Dutch speaker proficient in both English and German, and included two anglicists, three psychologists, and two cognitive neuroscientists. The reviewers were given written descriptions of the constructs intended to be measured and instructions for providing feedback regarding potential non-equivalence, ambiguity, and unintended meanings as well as suggestions for improvement. After each review round, the authors discussed the reviewers’ comments and revised items when deemed necessary. After two rounds of review, the task force had no further objections or suggestions for improvement. The final bilingual pool of 57 items is presented in Supplementary Table S1.

### Study procedure

Between October 2020 and March 2021, English- and German-speaking volunteers were invited to complete an anonymous cross-sectional online survey via invitations per email newsletters, social media posts, and word of mouth. These invitations led respondents to a landing page on the MIND Foundation’s website (https://www.mind-foundation.org/research/studies/) informing about the survey’s purpose in very general terms (“to improve our understanding of the acute and longer-term effects of psychedelics”). From here, those willing to participate were directed to a secure SoSci Survey (Leiner, 2019a) server hosted at Dresden University of Technology, Germany. The survey began with a request to choose between participation in English or German, followed by a consent form and assessment of inclusion criteria. To be allowed to complete the survey, participants had to (1) indicate a minimum age of 18 years, (2) endorse the ability to read, write, and speak the respective language fluently, (3) negate previous participation in the survey, and (4) endorse having felt at least once in their lifetime clearly discernible psychoactive effects after taking one of the following classic psychedelics: (1) LSD, (2) psilocybin or psilocybin-containing mushrooms, (3) mescaline or mescaline-containing cacti, or (4) ayahuasca. Participants who fulfilled these criteria were then allowed to complete the survey. To avoid potentially adverse attentional effects (see Hauser and Schwarz, 2015), no instructional manipulation checks were included in the survey. Participants were not compensated. The study was approved by the Institutional Review Board of Dresden University of Technology (SR-EK-355082020). The full survey is available upon request from the corresponding author.

### Study measures

#### Demographics

Demographic questions inquired about participants’ age, sex, country of residence, and level of education. The latter was assessed using the Comparative Analysis of Social Mobility in Industrial Nations (CASMIN) classification of education (Brauns et al., 2003) to ensure comparability between the English- and the German-language samples. For the sake of brevity, the two samples are referred to as the English sample and the German sample in the following. To characterize the samples in terms of mental health, participants were asked whether they had been diagnosed with a mental disorder in their lifetime, and if so, to indicate symptom area(s) associated with the given diagnosis or diagnoses.

#### Reports of selected psychedelic experiences

Participants were then asked to select one memorable psychedelic experience which they had undergone at least 1 month ago after taking one of the psychedelics listed in the inclusion criteria above, and which they were willing to report on in the remainder of the survey. Participants were explicitly informed that both positively and negatively evaluated experiences were eligible. After having chosen an experience to report on, participants were asked to indicate which psychedelic they had used, the time elapsed since the experience, the subjective clarity of their memory of the experience, the subjective strength of the dose, the subjective valence of the acute effects, any concomitant use of other psychoactive substances besides caffeine and nicotine, and psychedelic use before and after the reported experience.

##### Setting

To characterize the settings in which the reported experiences took place, participants were asked several dichotomous (yes/no) questions referring to specific setting categories (calm and undisturbed; familiar environment; nature or close-to-nature; party or festival; retreat; ceremonial; therapeutic). Participants were also asked to indicate whether or not a guide or sitter (i.e. a person whose task it was to support the participant during the experience, and who was not under the influence of any psychoactive substance at the time) was present, and to provide an estimate of the total number of people present during the reported experience.

##### Use motives

Motives for having the reported psychedelic experience were assessed following an example set by Haijen et al. (2018), but considering a broader and more differentiated set of potential intentions: Participants were presented with a list of 22 motives for using psychedelics (e.g. “out of curiosity,” “to treat psychological problems,” and “to have fun”), and were asked to rate the extent to which each item corresponded to their motives for undergoing the reported experiences at that time on a 4-point Likert-type scale (“not at all,” “somewhat,” “moderately,” and “very much”).

##### Characterization of the reported psychedelics experiences

In addition to the 57 original APEQ items, the following questionnaires were administered to characterize participants’ reported psychedelic experiences.

##### Emotional Breakthrough Inventory

The Emotional Breakthrough Inventory (EBI) is a recently validated six-item questionnaire designed to capture the “phenomenon of overcoming challenging emotions/memories and thereby experiencing emotional release or breakthrough” during psychedelic experiences (Roseman et al., 2019: 2). This definition bears close resemblance with our concept of relief explained above (i.e. the aspect complementary to distress within the “emotional experience of threat” domain). However, it should be noted that only two EBI items (items 4 and 6) correspond to the relief aspect of ACE in the narrower sense, whereas the remaining four items appear to relate more closely to the other two ACE aspects. Since there was to our knowledge no validated German version of the EBI at the time of data collection, we used our own translation of the instrument in the German version of the survey. Internal consistency of the EBI was high in both the English and the German sample (Cronbach’s alpha = 0.93 for both).

##### Challenging Experience Questionnaire

The Challenging Experience Questionnaire (CEQ; Barrett et al., 2016; German version by Dworatzyk et al., 2021) was developed as a multidimensional measure of different aspects of psychologically difficult experiences during the acute effects of psychedelics. The CEQ comprises 26 items and yields scores on seven subscales (*fear, grief, physical distress, insanity, isolation, death*, and *paranoia*) as well as a total scale, which was used in this study. Internal consistency of the total scale was very high in both the English and the German samples (Cronbach’s alpha = 0.95 for both).

#### Retrospective changes in psychological flexibility

The Acceptance and Action Questionnaire II (AAQ-II; Bond et al., 2011; German version by Hoyer and Gloster, 2013), measures psychological (in-)flexibility using seven items (e.g. “I am afraid of my feelings”) which are rated on a Likert-type scale from 1 (“never true”) to 7 (“always true”). Lower scores on the AAQ-II indicate greater psychological flexibility. In the present survey, we used an adapted version of the AAQ-II with statements phrased in past tense (e.g. “I was afraid of my feelings”) to assess psychological flexibility retrospectively (see Davis et al., 2020a, 2021). Each participant completed this adapted questionnaire with reference to the 3–4 weeks preceding (AAQ-II-before) and following the reported psychedelic experience (AAQ-II-after). The AAQ-II-before scale showed high internal consistency in both the English and the German samples (Cronbach’s alpha = 0.93 both). The AAQ-II-after scale also showed high internal consistency in both samples (Cronbach’s alpha = 0.93 and 0.89, respectively). Retrospective changes in psychological flexibility (AAQ-II-diff) were operationalized as the AAQ-II-after score subtracted from the AAQ-II-before score. Higher positive AAQ-II-diff scores thus indicate greater increases in psychological flexibility.

### Data analysis

#### Characteristics of participants and reported psychedelic experiences

After removing observations that were deemed invalid due to (1) speeding (scores ⩾2 on SoSci Survey’s TIME_RSI index; Leiner, 2019b), (2) using more than one psychedelic during the reported experience, (3) responding to the feedback request at the end of the survey in ways that raised concerns regarding the validity of reports, and/or (4) indicating poor memory of the reported experience, characteristics of the included participants and their reported psychedelic experiences were described and compared between the English and German samples.

#### Principal component analysis of use motives

Following the data analysis strategy reported by Haijen et al. (2018), we used principal component analysis (PCA) with orthogonal rotation (Varimax) to examine the factor structure underlying reported use motives in the complete sample of included participants. The primary aim of this was to compute component scores to be used as independent variables in a subsequent analysis of potential mediation effects (i.e. in to assess the assumption of context-dependence). PCA, which maximizes variance in lower-dimensional space, was therefore given preference over exploratory factor analysis as a dimension reduction strategy.

#### Confirmatory factor analyses of APEQ items

Confirmatory factor analyses (CFAs; using the software Mplus 8) were used for selecting the final APEQ items and for assessing the factor structure of the resulting final APEQ (model selection and replication). Since the APEQ is a theory-based instrument, these analyses served the initial validation of the APEQ as well as the verification of the first two of its overarching theoretical assumptions, that is, complementarity and intertwinedness. Because each item was specifically designed for one theory-derived subscale or ancillary scale, model selection was based solely on CFA and involved no exploratory factor analyses.

Model fit was assessed by evaluating multiple fit indices and by comparisons with simpler nested models. Following recommendations by Brown (2015), the Root-Mean Square Error of Approximation (RMSEA), the Confirmatory Fit Index (CFI), and the standardized root mean square residual (SRMR) were calculated as fit indices. Scaled *χ*^2^ difference tests (Satorra and Bentler, 2001) were used for nested model comparisons.

##### Matched strata for item/model selection and replication

To obtain independent participant samples for item/model selection and subsequent replication, the English sample and the German sample were each stratified into two subsamples matched on level of education, psychedelic used, subjective dose strength, and prior use of psychedelics. The following automated stratification procedure was carried out separately for both samples in Matlab (script available from corresponding author upon request): Observations from each cell of the factorial model assumed by the stratification variables were randomly assigned in equal parts to a “selection stratum” and a “replication stratum”. To avoid unnecessary discarding of valid data, residual observations from cells containing odd numbers of observations were grouped together and randomly assigned in equal parts to the two strata. Confirming the validity of the stratification procedure, *χ*^2^ independence tests showed no significant differences in the stratification variables between selection and replication strata in either the English or German sample.

##### Item selection

To ensure applicability of the APEQ in future research contexts with high demands for parsimony (e.g. psychometric batteries administered after psychedelic dosing sessions in clinical studies), we decided a priori that the final questionnaire should comprise no more than four items for each of the six subscales and two ancillary scales, that is, 32 items in total. As a basis for selecting the final four items for each subscales and ancillary scale, separate CFAs including one factor and all items in the respective subscale pool were calculated for the English and German selection strata. In the following item selection process, preference was given to items with relatively high and relatively similar factor loadings in both languages. In addition to these data-based criteria, item selection was guided by the motif that each subscale should, as far as possible, cover a broad range of acceptance/avoidance-related mental (i.e. cognitive, emotional, mnemonic, and sensory) events and processes. After item selection, a second iteration of CFAs was calculated to assess model fit, this time only including the four selected items of the given subscale.

##### Model selection and replication

To test the complete model, CFAs including all selected items, the eight first-order factors (avoidant response, accepting response, distress, relief, pro-avoidance insights, pro-acceptance insights, interaction, and introspection), and the two second-order factors AVE (representing the shared variance of the first-order factors avoidant response, distress, and pro-avoidance insights) and ACE (representing the shared variance of the first-order factors accepting response, relief, and pro-acceptance insights) were calculated for the English and German selection strata. These baseline models were then compared to a series of more constrained alternative models where two of the first-order factors were collapsed into one single factor, and *χ*^2^ difference tests of model fit were used to select the most parsimonious model. The selected measurement model was then replicated by repeating the same CFAs in the replication strata.

#### Structural equation model of potential mediation effects

The remaining two overarching theoretical assumptions, that is, context-dependence and interaction, were examined in the complete bilingual sample using structural equation modeling in Mplus 8. First, the selected and replicated measurement model (excluding the ancillary factors introspection and interaction) was calculated for the complete sample. To examine context-dependence, the measurement model was extended by regressing the second-order factors ACE and AVE on component scores extracted from the PCA of use motives. Furthermore, AAQ-II-diff scores indicating retrospective changes in psychological flexibility were regressed on these component scores, ACE and AVE. In order to test the assumption of interaction, the latent interaction term between ACE and AVE was also included in the model.

#### Correlation analyses

To further assess construct validity of the APEQ and examine theorized overlaps of ACE and AVE with the emotional breakthrough and challenging experience constructs, correlations of all APEQ scales with EBI and CEQ scores were calculated. To investigate how introspection and interaction with the environment relate to acceptance- and avoidance-related processes, correlations of the ancillary scales introspection and interaction with all ACE and AVE subscales were examined.

#### Derivation of a short form APEQ-S

To provide a brief measure of acceptance- and avoidance-related processes for research contexts with especially strong economy constraints, a 12-item short form (APEQ-S) comprising only the main scales ACE and AVE (without distinction between the subaspects) and omitting the ancillary scales introspection and interaction was derived. In order to achieve short form scales that are highly correlated with their long-form equivalents while also preserving the conceptual breath, two items from each ACE/AVE subaspect that were relatively highly correlated with the ACE/AVE sum score in both the English sample and the German sample were selected. Correlations of the APEQ-S with the long-form APEQ and external scales were examined.

## Results

### Participants

The survey URL was accessed by potential participants a total of 4790 times. Of the 3656 volunteers who agreed to participate, 1874 fulfilled the inclusion criteria and completed the entire survey. Out of these, 45 volunteers were excluded for one or more of the following reasons: Twenty-five volunteers reached scores ⩾2 on the TIME_RSI speeding index, sixteen volunteers indicated using more than one psychedelic during their reported experience, four volunteers’ free-entry responses to the feedback request at the end of the survey raised concerns regarding the validity of their reports, and one volunteer indicated that his memory of the reported experience was “not clear at all”.

Characteristics of the final sample of 1829 participants (997 in the English sample and 832 in the German sample) are presented in Table 1. Significant differences between English- and German-speaking participants were found for several characteristics, and moderate effect sizes were found for age, level of education, and lifetime diagnosis of anxiety disorder. Participants in the English sample reported 61 different countries of residence, and the most frequent mentions were the United States (45.3%), Germany (10.1%), Canada (7.4%), the United Kingdom (6.3%), the Netherlands (3.5%), Australia (2.3%), Portugal (1.7%), France (1.4%), Spain (1.2%), Greece (1.1%), Italy (1.1%), Brazil (1.0%), and Sweden (1.0%). Participants in the German sample reported 10 different countries of residence, and the most frequent mentions were Germany (94.4%), Austria (2.9%), and Switzerland (1.6%). All participants endorsed the ability to read, write, and speak the respective language fluently.

**Table 1.** Characteristics of included participants.

	Total sample (*N* = 1829)	English sample (*N* = 997)	German sample (*N* = 832)	*t* or *χ*^2^	*p*	Effect size (Cohen’s *d* or Cramer’s *V* or Cramer’s *φ*)
Mean (SD) age	30.4 (10.5)	32.0 (11.1)	28.6 (9.3)	−7.039	<0.001	−0.332
Sex				2.083	0.353	0.034
Male	67.4%	68.2%	66.5%			
Female	31.1%	30.0%	32.3%			
Other	1.5%	1.8%	1.2%			
CASMIN classification of education level^a^				190.881	<0.001	0.323
Tertiary education (highest)	66.0%	78.9%	50.6%			
Secondary education	32.1%	18.5%	48.6%			
Primary education (lowest)	1.8%	2.6%	0.8%			
Lifetime diagnosis of mental disorder	44.1%	53.3%	33.2%	73.501	<0.001	−0.200
Depression	33.4%	40.4%	25.0%	48.482	<0.001	−0.163
Anxiety	23.2%	34.8%	9.3%	166.249	<0.001	−0.301
Addiction	7.3%	9.2%	5.0%	11.669	0.001	−0.080
Mania	2.1%	2.9%	1.2%	6.331	0.012	−0.059
Psychosis	2.0%	1.8%	2.2%	0.301	0.581	0.013
Other	13.8%	15.5%	11.8%	5.402	0.020	−0.054

SD: standard deviation; CASMIN: comparative Analysis of Social Mobility in Industrial Nations (Brauns et al., 2003).

### Characteristics of reported psychedelic experiences

Characteristics of the psychedelic experiences reported by included participants are summarized in Table 2. The experiences reported by the English and German samples differed significantly with respect to several characteristics, but all of these comparisons showed only small effect sizes.

**Table 2.** Characteristics of the psychedelic experiences reported by included participants.

	Total sample (*N* = 1829)	English sample (*N* = 997)	German sample (*N* = 832)	*t* or *χ*^2^	*p*	Effect size (Cohen’s *d* or Cramer’s *V* or Cramer’s *φ*)
Mean (SD) years elapsed since experience	2.8 (5.6)	2.9 (6.2)	2.7 (4.7)	−0.742	0.458	−0.036
Subjective quality of memory^a^				37.851	<0.001	0.144
Completely clear	25.1%	29.6%	19.7%			
Very clear	42.5%	42.0%	43.1%			
Clear	25.2%	20.6%	30.8%			
Somewhat clear	7.2%	7.8%	6.4%			
Psychedelic used				65.988	<0.001	0.190
LSD	47.9%	39.3%	58.2%			
Psilocybin or psilocybin-containing mushrooms	42.5%	50.2%	33.3%			
Ayahuasca	8.4%	9.2%	7.3%			
Mescaline or mescaline-containing cacti	1.3%	1.3%	1.2%			
Subjective dose strength				15.987	0.003	0.093
Low	3.4%	4.0%	2.6%			
Moderate	39.3%	37.6%	41.2%			
High	39.0%	37.6%	40.6%			
Very high	14.8%	15.9%	13.3%			
Extremely high	3.6%	4.8%	2.2%			
Valence of acute effects				7.133	0.068	0.062
Rather pleasant	55.4%	54.1%	57.0%			
Rather unpleasant	3.9%	3.2%	4.8%			
Both pleasant and unpleasant	38.9%	40.5%	36.9%			
Neither pleasant nor unpleasant	1.8%	2.2%	1.3%			
Concomitant substance use						
None	60.3%	61.4%	58.9%	1.174	0.279	−0.025
Cannabis	31.8%	31.1%	32.6%	0.458	0.499	0.016
Alcohol	9.6%	8.4%	11.1%	3.614	0.057	0.044
Entactogens	2.6%	1.7%	3.7%	7.248	0.007	0.063
Stimulants	1.9%	1.5%	2.3%	1.509	0.219	0.029
Dissociatives	1.5%	1.4%	1.6%	0.078	0.780	0.007
Inhalants	0.5%	0.7%	0.2%	1.975	0.160	−0.033
Benzodiazepines	0.3%	0.5%	0.1%	2.017	0.156	−0.033
Opiates/opioids	0.2%	0.1%	0.2%	0.543	0.461	0.017
Other substance(s)	1.5%	1.4%	1.6%	0.078	0.780	0.023
Setting categories						
Calm, undisturbed environment	81.8%	81.7%	81.9%	0.003	0.953	0.001
Familiar environment	74.1%	77.5%	70.0%	13.575	<0.001	−0.086
Nature or close-to-nature environment	60.4%	55.0%	66.9%	27.227	<0.001	0.122
Setting designed for therapeutic purpose	11.5%	14.3%	8.1%	17.656	<0.001	−0.098
Party, concert, or festival	12.2%	9.3%	15.7%	17.378	<0.001	0.097
Psychedelic retreat	10.7%	9.7%	11.9%	2.232	0.135	0.035
Ceremonial, religious, or spiritual event	10.4%	10.9%	9.9%	0.562	0.453	−0.018
Presence of other people				49.048	<0.001	0.164
0 (alone)	20.4%	26.4%	13.3%			
2–5 people	59.4%	55.4%	64.3%			
6–15 people	11.3%	10.3%	12.5%			
16–30 people	2.8%	2.9%	2.8%			
31–100 people	2.1%	1.8%	2.4%			
>100 people	3.9%	3.2%	4.7%			
Guide/sitter present	30.8%	31.2%	30.4%	0.131	0.717	−0.008
Number of days with psychedelic use prior to reported experience				57.497	<0.001	0.177
0 (never before)	25.9%	24.6%	27.4%			
1–5	25.9%	21.7%	31.0%			
6–20	18.6%	17.2%	20.4%			
21–50	12.1%	14.2%	9.5%			
51–100	8.4%	10.7%	5.5%			
>100	9.1%	11.6%	6.1%			

SD: standard deviation.

a There was one participant who indicated his memory of the reported psychedelic experience was “not clear at all.” This participant was excluded; hence, frequencies for this response option are not reported here.

#### Use motives

Bartlett’s test of sphericity (*χ*^2^(231) = 11,948.748; *p* < 0.001) and the Kaiser–Mayer–Olkin measure of sampling adequacy (0.842) indicated that participants’ responses to the use motives items were suitable for PCA. Five components with eigenvalues greater than 1 were found using PCA, but the scree plot suggested a three-component solution was appropriate. The three components cumulatively explained 44.2% of the variance. Based on the loadings listed in Table 3, the components were named (1) “therapeutic intention”, (2) “escapist intention”, and (3) “hedonic intention”. For each component and participant, component scores were extracted to be entered as independent variables in the structural equation model (SEM) reported below exploring potential mediation effects. Whereas component scores of English- and German-language participants did not differ significantly in terms of escapist (*t*(1,827) = −0.875; *p* = 0.381) and hedonic intentions (*t*(1,827) = 0.207; *p* = 0.836), therapeutic intentions were much less pronounced in the German sample (*t*(1,827) = −17.964; *p* < 0.001; Cohen’s *d* = −0.848).

**Table 3.** Item loadings from the principal component analysis (PCA) of use motives in the complete bilingual sample (*N* = 1829).

Item	Component 1: “Therapeutic intention”	Component 2: “Escapist intention”	Component 3: “Hedonic intention”
To treat psychological problems	**0.780**	−0.077	0.027
To confront difficult feelings	**0.737**	−0.248	0.038
To escape from difficult feelings	**0.636**	0.443	−0.035
To increase my well-being	**0.573**	−0.133	0.371
To increase my performance	**0.527**	−0.049	0.406
To treat physical problems	**0.492**	−0.051	0.085
To spend time with friends	**−0.475**	0.316	0.451
Out of curiosity	**−0.289**	0.103	0.062
To distract myself from problems	0.402	**0.662**	0.049
Out of boredom	0.017	**0.585**	0.104
For personal growth	0.495	**−0.576**	0.235
To intoxicate myself	−0.335	**0.547**	0.207
To have fun	−0.480	**0.524**	0.419
For spiritual reasons	0.338	**−0.506**	0.341
For partying	−0.306	**0.505**	0.270
For self-awareness	0.425	**−0.503**	0.359
To fit in	−0.072	**0.332**	−0.044
For religious reasons	0.249	**−0.271**	0.254
To increase my creativity	0.090	−0.084	**0.715**
To have an experience of nature	−0.150	−0.168	**0.612**
For relaxation	0.060	0.286	**0.599**
To increase sexual pleasure	0.132	0.103	**0.392**

Items were rated on a 4-point Likert-type scale (“not at all”, “somewhat,” “moderately,” and “very much”).

The highest loading of each item is written in bold font.

### Item selection

Factor loadings and model fit indicators for the CFAs performed for item selection in the English (*n* = 498) and German selection stratum (*n* = 416) are summarized in Supplementary Table S1 and Supplementary Table S2, respectively. After item selection, model fit was good for all subscales in both selection strata. The final selection of 32 items and internal consistencies for all scales, subscales, and ancillary scales are presented in Table 4.

**Table 4.** Descriptive statistics and internal consistencies for the final selection of 32 APEQ items and 12 APEQ-S items, scales, subscales, and ancillary scales in the complete English (*N* = 997) and German sample (*N* = 832).

Scale	Item # in original item pool/English item text (items selected for APEQ-S in bold font)	Mean (SD)		*t*	*p*	Cohen’s *d*	Cronbach’s alpha for APEQ (APEQ-S)	
		English	German				English	German
Acceptance-related experience (ACE)		63.9 (23.2)	53.9 (24.7)	8.887	<0.001	0.417	0.92 (0.87)	0.93 (0.88)
Accepting response		61.5 (25.6)	52.4 (28.0)	7.239	<0.001	0.339	0.79	0.86
19. I was able to accept uncomfortable thoughts or memories.		65.1 (33.8)	59.9 (30.3)					
25. I was open to difficult sensations or emotional states.		71.2 (28.7)	58.6 (32.8)					
26. **I looked at painful memories with openness.**		57.4 (34.4)	49.0 (35.7)					
53. **I managed to confront a personal fear.**		52.4 (36.1)	42.3 (34.5)					
Relief		68.8 (24.7)	59.4 (29.4)	7.780	<0.001	0.339	0.83	0.84
6. **It seemed to me as if some kind of blockage was being resolved.**		64.4 (33.7)	55.9 (35.4)					
16. **I had a positive emotional breakthrough.**		72.5 (29.1)	58.7 (33.5)					
23. I felt a sense of relief.		69.4 (30.3)	65.6 (29.9)					
40. Things became easier for me in a liberating way.		68.8 (28.5)	57.3 (32.8)					
Pro-acceptance insights		61.4 (26.3)	50.0 (27.4)	9.043	<0.001	0.424	0.85	0.85
24. **I learned to better understand certain emotional states.**		68.5 (29.3)	61.1 (24.4)					
44. **I discovered a deeper acceptance of certain difficult feelings or sensations.**		65.3 (31.5)	52.2 (34.0)					
50. I noticed that certain thoughts or memories are not as dangerous for me as I had previously thought.		54.6 (33.6)	44.2 (33.3)					
54. I learned to appreciate certain uncomfortable feelings or sensations more.		57.4 (32.6)	42.7 (32.6)					
Avoidance-related experience (AVE)		21.9 (20.1)	18.3 (20.3)	3.822	<0.001	0.178	0.89 (0.81)	0.93 (0.86)
Avoidant response		26.5 (23.9)	22.7 (23.8)	3.445	0.001	0.159	0.78	0.84
14. **I tried to lessen, or rid myself of, certain perceptions or bodily sensations.**		33.2 (33.0)	23.4 (30.3)					
18. I tried to change my mood.		25.7 (30.1)	25.1 (29.6)					
46. **I attempted to suppress certain emotions or thoughts.**		20.8 (28.5)	19.3 (27.2)					
51. I made efforts to avoid or control difficult feelings.		26.4 (30.8)	22.9 (28.9)					
Distress		22.5 (27.6)	17.6 (24.7)	3.957	<0.001	0.176	0.90	0.83
27. I felt tormented.		20.9 (31.0)	17.6 (28.2)					
39. **I panicked.**		18.9 (29.4)	16.3 (27.8)					
45. I experienced a state of distress.		29.6 (34.6)	20.4 (29.1)					
55. **I suffered from what I was experiencing.**		20.7 (30.3)	16.3 (27.9)					
Pro-avoidance insights		16.6 (19.1)	14.5 (19.1)	2.343	0.019	0.110	0.70	0.79
15. **I learned to fear or detest certain uncomfortable feelings or sensations more strongly.**		17.5 (27.5)	14.3 (24.0)					
36. I noticed that I can tolerate certain mental states less than I thought.		19.8 (28.0)	18.0 (26.9)					
37. I learned that it is better for me not to experience certain emotional states at all.		13.2 (23.7)	11.6 (22.1)					
57. **I learned that certain thoughts or memories are more dangerous for me than I previously thought.**		15.9 (25.6)	14.1 (24.4)					
Ancillary scales								
Introspection		76.1 (21.3)	66.6 (24.0)	8.955	<0.001	0.419	0.84	0.87
2. I was engaged with what was going on inside me.		82.6 (21.9)	75.1 (24.8)					
12. I looked inside.		79.1 (25.5)	69.0 (27.2)					
30. My attention was turned inward.		71.2 (27.4)	61.1 (29.0)					
43. I was absorbed in my inner experience.		71.5 (28.4)	61.1 (31.8)					
Interaction		63.9 (24.7)	67.3 (24.5)	2.977	0.003	0.139	0.75	0.80
1. I observed my external environment.		70.4 (29.7)	73.3 (27.9)					
22. I actively engaged with my surroundings.		59.2 (33.2)	63.3 (32.9)					
48. I interacted with other people.		55.1 (37.6)	62.4 (34.3)					
49. I moved my body.		70.9 (29.4)	69.9 (28.0)					

SD: standard deviation; APEQ: Acceptance/Avoidance-Promoting Experiences Questionnaire.

Note that the APEQ-S includes the main scales ACE and AVE only. Paper and pencil versions of the APEQ and the APEQ-S in English and German, including the final order of items, are provided in the Supplementary Materials and at https://mind-foundation.org/research/resources/.

APEQ-S items are written in the bold font.

### Model selection and replication

Table 5 summarizes fit statistics for the baseline models and alternative models as well as scaled *χ*^2^ difference tests for model comparisons. The baseline model showed acceptable fit in both the English (RMSEA = 0.052 (90% confidence interval (CI) = 0.048–0.056); CFI = 0.912; SRMR = 0.062) and the German selection stratum (RMSEA = 0.050 (90% CI = 0.046–0.055); CFI = 0.927; SRMR = 0.074). However, a more constrained alternative model where the first-order factors accepting response and pro-acceptance insights were collapsed also showed acceptable fit in English (RMSEA = 0.052 (90% CI = 0.048–0.056); CFI = 0.911; SRMR = 0.062) and German (RMSEA = 0.050 (90% CI = 0.046–0.055); CFI = 0.926; SRMR = 0.074). Compared to the baseline model, this model’s fit to the data was not significantly worse in both selection strata (*χ*^2^_diff_ = 5.9; *p* = 0.051 and *χ*^2^_diff_ = 5.6; *p* = 0.060, respectively), indicating that the two first-order factors accepting response and pro-acceptance insights were statistically hard to distinguish in both samples. The more parsimonious alternative model where the two factors were collapsed into a single factor was therefore selected for further analyses.

**Table 5.** Model comparisons in the English and German selection strata.

Sample/model	Model fit						Fit vs baseline model		
	*χ* ^2^	df	*p*	RMSEA	CFI	SRMR	*χ* ^2^_diff_	df	*p*
English selection stratum (*n* = 498)									
Baseline model	1052.9	452	<0.001	0.052	0.912	0.062			
Accepting response = breakthrough	1184.1	454	<0.001	0.057	0.893	0.064	98.8	2	<0.001
Accepting Response = pro-acceptance insights	1059.1	454	<0.001	0.052	0.911	0.062	5.9	2	0.051
Relief = pro-acceptance insights	1174.2	454	<0.001	0.056	0.895	0.063	90.1	2	<0.001
Avoidant response = distress	1155.5	454	<0.001	0.056	0.897	0.066	102.2	2	<0.001
Avoidant response = pro-avoidance insights	1089.3	454	<0.001	0.053	0.907	0.063	22.4	2	<0.001
Distress = pro-avoidance insights	1232.0	454	<0.001	0.059	0.886	0.065	59.5	2	<0.001
German selection stratum (*n* = 416)									
Baseline model	929.5	452	<0.001	0.050	0.927	0.074			
Accepting response = relief	1066.8	454	<0.001	0.057	0.906	0.075	81.8	2	<0.001
Accepting response = pro-acceptance insights	935.3	454	<0.001	0.050	0.926	0.074	5.6	2	0.060
Relief = pro-acceptance insights	1059.8	454	<0.001	0.057	0.907	0.075	87.4	2	<0.001
Avoidant response = distress	1046.0	454	<0.001	0.056	0.909	0.075	50.0	2	<0.001
Avoidant response = pro-avoidance insights	949.1	454	<0.001	0.051	0.924	0.074	14.1	2	<0.001
Distress = pro-avoidance insights	983.2	454	<0.001	0.053	0.919	0.077	26.4	2	<0.001

RMSEA: root-mean square error of approximation; CFI: Comparative Fit Index; SRMR: standardized root mean residual.

**Figure 2.**
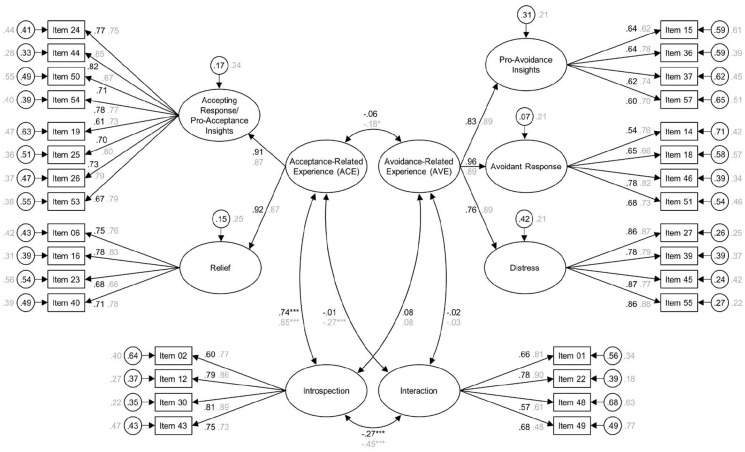
Summary of the selected measurement model in the English (black font; *n* = 499) and German replication stratum (gray font; *n* = 416). Ellipses represent the latent variables, and rectangles represent the manifest variables. Numbers next to long straight arrows are factor loadings. Circled numbers next to short straight arrows are residual variances. Numbers next to curved double-headed arrows are correlations among latent variables. All coefficients are standardized. The first-order factors accepting response and pro-acceptance insights were collapsed into one single factor since they were statistically hard to distinguish in both selection strata (*p* = 0.051 and *p* = 0.060, respectively). In accordance with the assumption of complementarity, the correlation between the second-order factors ACE and AVE was non-significant in the English sample (*r* = −0.06; *p* = 0.421) and significantly negative but weak in the German sample (*r* = −0.18; *p* = 0.014).

The selected measurement model was then validated by repeating the same CFAs in the English (*n* = 498) and the German replication stratum (*n* = 416). Figure 2 provides summaries of this model for both languages. Model fit was acceptable in both the English (RMSEA = 0.047 (90% CI = 0.043–0.051); CFI = 0.923; SRMR = 0.066) and the German replication stratum (RMSEA = 0.056 (90% CI = 0.051–0.060); CFI = 0.911; SRMR = 0.076). In accordance with the theorized assumption of complementarity, the correlation between the second-order factors ACE and AVE was non-significant in the English sample (*r* = −0.06; *p* = 0.421) and significantly negative but weak in the German sample (*r* = −0.18; *p* = 0.014). As expected, there was a strong positive correlation between ACE and the first-order factor corresponding to the ancillary scale introspection in both languages (*r* = 0.74; *p* < 0.001 and *r* = 0.65; *p* < 0.001, respectively). The correlation between ACE and the first-order factor corresponding to the ancillary scale interaction was non-significant in the English sample (*r* = −0.01; *p* = 0.824) but significantly negative in the German sample (*r* = −0.27; *p* < 0.001, respectively). Correlations of AVE with introspection (*r* = 0.08; *p* = 0.196 and *r* = 0.09; *p* = 0.122 in the English and German samples, respectively) and interaction (*r* = −0.02; *p* = 0.710 and *r* = −0.03; *p* < 0.664, respectively) were non-significant in both languages. The correlation between introspection and interaction was significantly negative in both languages (*r* = −0.27; *p* < 0.001 and *r* = −0.45; *p* < 0.001, respectively).

### SEM of potential mediation effects

**Figure 3.**
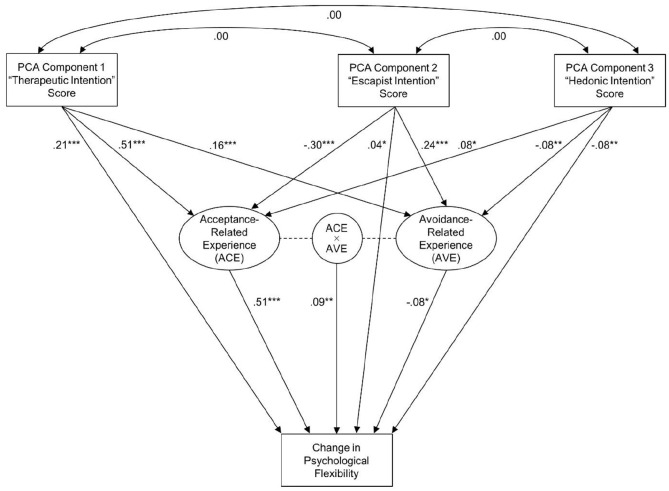
Summary of the structural equation model (SEM) investigating potential mediation effects, associations between use motives, acute acceptance-related experience (ACE) and avoidance-related experience (AVE), and retrospective longer-term changes in psychological flexibility in the complete bilingual sample (*N* = 1829). Ellipses represent the second-order latent variables ACE and AVE, and the circle over the dashed line between them represents their latent interaction term. Rectangles represent manifest variables. Numbers next to straight arrows are regression weights. Numbers next to curved double-headed arrows are correlations. All coefficients are standardized.

**Figure 4.**
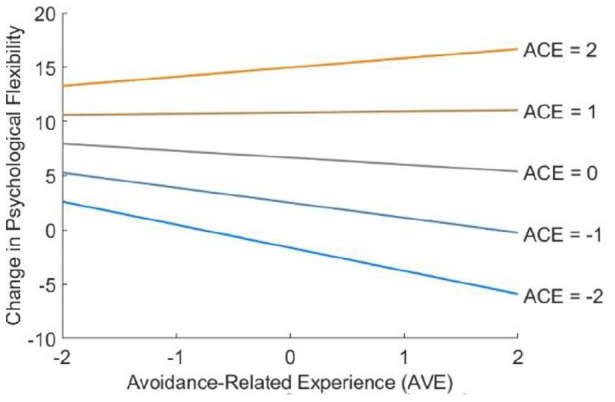
Associations of the latent second-order factors ACE (*β* = 4.145; *p* < 0.000), AVE (*β* = −0.638; *p* = 0.013), and their latent interaction term (*β* = 0.745; *p* = 0.005) with retrospective reports of change in psychological flexibility according to the structural equation model (SEM) summarized in Figure 3. Unstandardized coefficients are used here for better interpretability. The scale of the independent variables ACE and AVE can be treated as standardized nonetheless, since their mean and variance were fixed at 0 and 1, respectively.

Calculating the selected and replicated measurement model (without the ancillary factors) in the complete bilingual sample yielded acceptable fit indices (RMSEA = 0.051 (90% CI = 0.049–0.054); CFI = 0.936; SRMR = 0.063). The subsequent SEM assessing potential mediation effects is summarized in Figure 3. In line with the assumption of context-dependence, ACE was positively associated with therapeutic intention (*β* = 0.507; *p* < 0.001) and hedonic intention (*β* = 0.165; *p* < 0.001), and negatively associated with escapist intention (*β* = −0.300; *p* < 0.001). AVE was positively associated with therapeutic intention (*β* = 0.157; *p* < 0.001) and escapist intention (*β* = 0.238; *p* < 0.001), and negatively associated with hedonic intention (*β* = −0.078; *p* = 0.002). Retrospective changes in psychological flexibility were positively associated with ACE (*β* = 0.500; *p* < 0.001) and negatively associated with AVE (*β* = −0.077; *p* = 0.013). In addition to these main effects, and in line with the assumption of interaction, there was a significant interaction effect between ACE and AVE (*β* = 0.090; *p* = 0.005): As illustrated in Figure 4, the negative effect of AVE on changes in psychological flexibility was more pronounced when ACE was low. Apart from its indirect associations with changes in psychological flexibility (via ACE and AVE), therapeutic intention also had a positive direct effect (*β* = 0.207; *p* < 0.001), indicating that the association between therapeutic intention and changes in psychological flexibility was not entirely attributable to ACE and AVE. In contrast, the direct effect of escapist intention was statistically non-significant (*β* = 0.041; *p* = 0.065), indicating that its association with changes in psychological flexibility was entirely attributable to ACE and AVE. The direct effect of hedonic intention was significantly negative (*β* = −0.083; *p* < 0.001), and thus in the opposite direction as both of its indirect effects. The sum of both indirect effects was similar in size to the antagonistic direct effect, indicating the presence of a suppression effect (MacKinnon et al., 2000).

### Associations with external scales

Intercorrelations between sum scores on APEQ main scales, subscales, and ancillary scales as well as correlations with external scales in the complete bilingual sample are shown in Table 6. In accordance with the assumption of intertwinedness, subscales within the main scales ACE and AVE showed strong positive correlations among each other. Supporting construct validity, strong positive correlations were found between ACE and EBI scores (*r* = 0.81, *p* < 0.001) and also between AVE and CEQ scores (*r* = 0.75, *p* < 0.001).

**Table 6.** Intercorrelations between APEQ main scales, subscales, and ancillary scales and correlations with external scales in the complete bilingual sample (*N* = 1829).

	*r* (*p*)											
	ACE	Accepting response	Relief	Pro-acceptance insights	AVE	Avoidant response	Distress	Pro-avoidance insights	Introspection	Interaction	EBI	CEQ
APEQ main scales/subscales												
ACE	–											
Accepting response	0.92 (<0.001)	–										
Relief	0.86 (<0.001)	0.66 (<0.001)	–									
Pro-acceptance insights	0.93 (<0.001)	0.83 (<0.001)	0.70 (<0.001)	–								
AVE	−0.03 (0.213)	0.02 (0.527)	−0.16 (<0.001)	0.06 (0.013)	–							
Avoidant response	−0.07 (0.003)	−0.04 (0.071)	−0.17 (<0.001)	0.02 (0.476)	0.89 (<0.001)	–						
Distress	0.00 (0.943)	0.07 (0.004)	−0.13 (<0.001)	0.06 (0.009)	0.89 (<0.001)	0.66 (<0.001)	–					
Pro-avoidance insights	−0.01 (0.761)	0.01 (0.797)	−0.11 (<0.001)	0.08 (0.001)	0.84 (<0.001)	0.65 (<0.001)	0.61 (<0.001)	–				
APEQ ancillary scales												
Introspection	0.60 (<0.001)	0.57 (<0.001)	0.50 (<0.001)	0.57 (<0.001)	0.12 (<0.001)	0.06 (0.020)	0.17 (<0.001)	0.08 (<0.001)	–			
Interaction	−0.09 (<0.001)	−0.12 (<0.001)	−0.07 (0.004)	−0.06 (0.006)	−0.09 (<0.001)	0.00 (0.896)	−0.18 (<0.001)	−0.02 (0.373)	−0.30 (<0.001)	–		
External scales												
EBI	0.81 (<0.001)	0.76 (<0.001)	0.68 (<0.001)	0.76 (<0.001)	0.12 (<0.001)	0.06 (0.013)	0.16 (<0.001)	0.11 (<0.001)	0.55 (<0.001)	−0.21 (<0.001)	–	
CEQ	0.09 (<0.001)	0.15 (<0.001)	−0.05 (0.037)	0.14 (<0.001)	0.75 (<0.001)	0.60 (<0.001)	0.82 (<0.001)	0.53 (<0.001)	0.24 (<0.001)	−0.21 (<0.001)	0.23 (<0.001)	–

ACE: acceptance-related experience; AVE: avoidance-related experience; EBI: Emotional Breakthrough Inventory; CEQ: Challenging Experience Questionnaire.

### Associations of the ancillary scales introspection and interaction with subaspects of ACE and AVE

As expected, the ancillary scale introspection was positively correlated with all ACE subscales (accepting response: *r* = 0.57, *p* < 0.001; relief: *r* = 0.50, *p* < 0.001; pro-acceptance insights: *r* = 0.57, *p* < 0.001) in the complete bilingual sample (see Table 6). Also as hypothesized, the ancillary scale interaction was negatively correlated with the AVE subscale distress (*r* = −0.18, *p* < 0.001). However, the hypothesized positive correlations between interaction and the remaining AVE subscales were not found in the complete bilingual sample. Since the hypothesis that higher levels of interaction are differentially associated with AVE subaspects (less distress, but more avoidant responding and more pro-avoidance insights) was formulated for specific use contexts resembling psychedelic-assisted therapy rather than psychedelic use in general, we repeated the analysis in an exploratory manner in a subset of observations where participants indicated that the experience had taken place in a calm and undisturbed environment (aiming to exclude cases where high levels of interaction are likely due to situational demands rather than avoidance). Furthermore, participants with below-average therapeutic intentions, identified based on the PCA of use motives (scores below 0 on the first component), were excluded.

In the resulting subsample, which included 742 observations (534 English and 208 German-language participants), the ancillary scales introspection and interaction were again negatively correlated with one another (*r* = −0.19, *p* < 0.001). Like in the complete sample, the introspection scale was positively correlated with all ACE subscales (accepting response: *r* = 0.50, *p* < 0.001; relief: *r* = 0.42, *p* < 0.001; pro-acceptance insights: *r* = 0.49, *p* < 0.001). The interaction scale was not significantly correlated with the ACE subscales accepting response (*r* = 0.01; *p* = 0.712) and relief (*r* = 0.04, *p* = 0.229), but significantly positively correlated with the pro-acceptance insights subscale (*r* = 0.12, *p* = 0.002). Although correlations with AVE subscales were generally weak and would likely not have reached statistical significance in a smaller sample, they showed the hypothesized differential pattern: The interaction scale was positively correlated with the subscales avoidant response (*r* = 0.15, *p* < 0.001) and pro-avoidance insights (*r* = 0.12, *p* = 0.001) but negatively correlated with the distress subscale (*r* = −0.09, *p* = 0.020). Conversely, the introspection scale was negatively correlated with the avoidant response subscale (*r* = −0.10, *p* = 0.008) and positively correlated with the distress subscale (*r* = 0.10, *p* = 0.007). The correlation between the introspection scale and the pro-avoidance insights subscale was non-significant (*r* = −0.04, *p* = 0.306).

### Short form APEQ-S

Table 4 shows the 12 items selected for the APEQ-S (written in bold font) and internal consistencies in the complete English and German samples. Correlations between the APEQ-S and the APEQ were very strong in both samples for ACE (*r* = 0.97, *p* < 0.001 both) and AVE (*r* = 0.96, *p* < 0.001 and *r* = 0.97, *p* < 0.001, respectively). In accordance with the assumption of complementarity, and in line with results for the long-form APEQ, the correlation between ACE and AVE in the APEQ-S was non-significant in the English sample (*r* = 0.06, *p* = 0.063) and significantly negative but weak in the German sample (*r* = −0.09, *p* = 0.011).

Like the respective scores from the long-form APEQ, ACE scores from the APEQ-S were positively correlated with the EBI (*r* = 0.83, *p* < 0.001) and slightly positively correlated with the CEQ (*r* = 0.14, *p* < 0.001) in the complete bilingual sample. AVE scores from the APEQ-S were slightly positively correlated with the EBI (*r* = 0.12, *p* < 0.001) and positively correlated with the CEQ (*r* = 0.74, *p* < 0.001).

## Discussion

This study was conducted in order to validate a newly developed research instrument for investigating acceptance- and avoidance-promoting effects of psychedelic drugs, the APEQ, and to empirically examine the psychological model that forms its theoretical basis. The components of the theorized model and its overarching assumptions were largely confirmed. Evidence supporting the model’s assumptions of complementarity and intertwinedness was found in CFAs assessing the factor structure of the APEQ. A subsequent SEM in the complete bilingual sample modeling associations of ACE and AVE with use motives and retrospective reports of changes in psychological flexibility provided evidence supporting the assumptions of context-dependence and interaction. Convergent associations of AVE and ACE with CEQ and EBI scores, respectively, lend further support to the construct validity of the APEQ, and add some clarity to the role of challenging (Barrett et al., 2016) and emotional breakthrough experiences (Roseman et al., 2019) in determining longer-term responses to psychedelics.

CFAs showed a high degree of convergence between the independent English and German samples, suggesting that the present results are highly generalizable. Compared to the German sample, the English sample scored significantly higher on ACE, AVE, and all subscales. Regarding the ancillary scales, the English sample scored significantly higher on introspection and lower on interaction. A possible explanation for these differences is the fact that participants in the English sample more often reported lifetime diagnoses of mental disorders, especially in the domain of anxiety disorders. Correspondingly, English-language participants indicated much stronger therapeutic intentions for psychedelic use, which can explain that their reported experiences were more therapeutic (in terms of ACE) but also more challenging.

### Evidence of complementarity and intertwinedness

Development of the APEQ was based on the central premises of complementarity (the assumption that contrasting acceptance- and avoidance-related experiential aspects cannot occur simultaneously but may alternate over the course of a psychedelic experience) and intertwinedness (the assumption that distinguishable subaspects within the acceptance- and avoidance-related domain typically co-occur and correspond to one another in magnitude). In accordance with the assumption of complementarity, we found that ACE and AVE were indeed not only distinguishable but also largely independent factors. In contrast, and in line with the assumption of intertwinedness, subaspects within ACE and AVE were found to be highly positively correlated: Levels of distress were strongly associated with avoidant responding and pro-avoidance insights. Likewise, experiences of relief were strongly associated with accepting responses and pro-acceptance insights. Associations between the latter two were in fact so strong that the baseline model and a model where both were collapsed into one factor fit the data comparably well in both selection strata. Note, however, that the non-significance of *χ*^2^ difference tests for model comparisons was indeed marginal in both samples. Therefore, and based on the theoretical consideration that accepting responses to aversive private events must not always inevitably lead to insights that promote acceptance (e.g. when acceptance-promoting beliefs with respect to the same private events have already been held previously), we propose to maintain both subscales and further investigate their dissociability in future prospective studies.

### Evidence of context-dependence

Like most extra-pharmacological conceptions of acute and longer-term psychedelic drug effects (Carhart-Harris and Nutt, 2017), the present theorized model (Figure 1) postulates a mediation effect: Context factors determine the acute drug response, which in turn predicts longer-term outcomes. While the present cross-sectional and retrospective data collection method does not technically allow for the identification of mediation effects (for an in-depth discussion of mediation analysis in psychedelic research, see Kangaslampi, 2020), the SEM summarized in Figure 3 is consistent with the mediation assumption and may provide preliminary evidence that ACE and AVE are potential mediators between different motives for using psychedelics and longer-term changes in psychological flexibility. In line with the view that learning acceptance requires a willingness to confront and engage with personally meaningful negative emotions, ACE was strongly positively associated with therapeutic intentions. Conversely, escapist intentions (e.g. using psychedelics out of boredom or for distraction from personal problems) were negatively associated with ACE and positively associated with AVE. This is compatible with the idea that an avoidant mind-set can decrease the likelihood of spontaneously relinquishing avoidance in the face of distress, thereby impeding operant shaping processes of “learning to let go” that would otherwise facilitate the resolution of challenging experiences (Wolff et al., 2020). That therapeutic intentions were also positively (but less strongly) associated with AVE is not surprising, considering that the willingness to confront strong negative emotions should by no means imply that one is also capable of doing so without difficulty from the outset.

It is noteworthy that, compared to escapist intentions, hedonic intentions showed an opposite pattern of associations with ACE and AVE. This result adds nuance to a previous finding linking recreational psychedelic use with lower rates of challenging experiences (Haijen et al., 2018), and suggests that the distinction between approach-motivated (hedonic) and avoidance-motivated (escapist) recreational use could be important for understanding and preventing psychedelic-induced harm. One particular form of avoidance-motivated behavior with high potential relevance for psychedelic harm reduction is the phenomenon of *spiritual bypass*, that is, the tendency to avoid or prematurely transcend emotional issues, developmental tasks, and basic human needs by focusing on spiritual beliefs, practices, and experiences (Gorman et al., 2021; Welwood, 1984). The spiritual or mystical dimension of the psychedelic experience has received much attention as a potential source of positive change in clinical applications and beyond (Aday et al., 2021). However, at least in some cases, spiritual or mystical-type psychedelic experiences could play the more negative role of motivating continued psychedelic-assisted spiritual bypass. In the light of the present results, which suggest avoidance-motivated psychedelic experiences can further reduce psychological flexibility (see discussion below), escalation of spiritual bypass by repeated psychedelic use appears to be possible.

### Evidence of interaction

The idea that ACE and AVE are potential mediators of psychedelic-induced longer-term change is further supported by the finding that retrospective reports of changes in psychological flexibility were positively associated with ACE and negatively associated with AVE. Crucially, and in line with the assumption of interaction, there was also a positive interaction effect. As illustrated in Figure 4, a clear negative effect of AVE on changes in psychological flexibility was seen among cases where ACE was relatively low, but was absent when ACE was relatively high. This observation is consistent with the idea that AVEs can promote experiential avoidance in the longer term, but only in the relative absence of complementary acceptance-related processes. Accordingly, even desperate resistance and intense distress are not necessarily detrimental to beneficial longer-term outcomes when the same experience is also characterized by accepting responses, experiences of relief, and corresponding pro-acceptance insights. Qualitative studies of patient reports seem to indicate that the succession of initial distress and subsequent relief may be a rather typical course of events in therapeutically effective psychedelic dosing sessions (Belser et al., 2017; Gasser et al., 2015; Watts et al., 2017). Extrapolating from a well-established finding of process-outcome psychotherapy research, a certain discomfort associated with the general change mechanism *problem activation* (Grawe, 1997) may in fact be necessary for corrective acceptance-promoting experiences to occur. Almost all conceptualizations of psychotherapy share the view that, in order to overcome their emotional problems, patients must come into direct contact with them (Frank, 1961; Grawe, 1997; Orlinsky et al., 1994), that is, “the only way out is through” (Pascual-Leone and Greenberg, 2007). Referring to psychedelic-assisted therapies, Carhart-Harris et al. (2018b) concurred that “challenging experiences can indeed be therapeutically beneficial, but only if personal insight and/or an emotional catharsis follows the relevant experience of psychological struggle”. In accordance with this conditional statement, previous studies have reported mixed results regarding longer-term outcomes of challenging psychedelic experiences. Roseman et al. (2018) found that levels of anxiety and confusion during psilocybin sessions predicted less positive clinical outcomes in depression patients. Likewise, a prospective survey study with recreational psychedelic users found that challenging experiences were negatively associated with subsequent well-being (Haijen et al., 2018). However, another survey found that well-being was negatively related to the duration of previous challenging experiences but positively related to their intensity (Carbonaro et al., 2016). Further evidence that longer-term impacts of challenging psychedelic experiences depend on additional factors was recently presented by Davis et al. (2021). In a multiple regression model controlling for the insightfulness of psychedelic experiences, these authors found a significantly negative association between CEQ scores and retrospective changes in psychological flexibility, but this association was not seen in a simpler regression model that did not control for insightfulness. Taken together, these mixed findings suggest that the challenging experience construct underlying the CEQ, which does not distinguish between resolved and unresolved instances of distressful experiences, is—when taken by itself—only limitedly useful for predicting psychedelic-induced longer-term changes. Roseman et al. (2019) developed the EBI to fill this gap and provide a measure of “overcoming challenging emotions/memories and thereby experiencing emotional release or breakthrough.” An important detail regarding the EBI is that the authors discarded two negatively worded items from the final scale after finding that these items did not load on the same factor as the six positively worded items. Given our present results, it can be suspected that the (maintained) first EBI factor is closely related to ACE whereas the (discarded) second factor was akin to AVE (and thus, the challenging experience construct). In line with this, here we have provided evidence that ACE and AVE are not opposing ends of a single dimension but two relatively independent, complementary aspects of the psychedelic experience whose interplay is critical for longer-term benefits and harms associated with psychedelic use. Based on this, we not only caution against disregarding one or the other aspect, but also stress the importance of considering the crucial interaction between the two when investigating longer-term effects of psychedelics on psychological flexibility and related outcomes.

### On the roles of introspection and interaction with the environment

The two ancillary scales introspection and interaction differ from the APEQ main scales in that the complementary constructs they are designed to measure are not defined in relation to aversive private events, and therefore not straightforwardly associated with acceptance- and avoidance-related processes. The choice of placing one’s focus of engagement either more internally or rather on the external environment is usually given irrespective of whether negative emotions and associated sensations, thoughts, or memories are absent or present. In the latter case, adding complexity, interaction with the environment can be a form of accepting engagement (e.g. describing aversive imagery to another person) but also an act of avoidance (e.g. walking and looking around to tone down aversive imagery via active inference and well-defined sensory input; Pink-Hashkes et al., 2017; Wolff et al., 2020). In accordance with the former possibility, the interaction scale was positively correlated with pro-acceptance insights in a subset of experiences where context in terms of set and setting approximately resembled psychedelic-assisted therapy. Supporting the latter possibility, and as hypothesized, the interaction scale was significantly positively correlated with the AVE subscales avoidant response and pro-avoidance insights, but significantly negatively correlated with the distress subscale. This result could be theoretically important as it points to the possibility that avoidance-promoting psychedelic experiences are not necessarily always experienced (or remembered) as distressful, which may be relevant for certain harm scenarios, such as the above-mentioned escalation of spiritual bypass by repeated avoidance-motivated psychedelic use.

That we found a converse differential pattern of correlations with the introspection scale may further support the view that switching from an introspective state to interacting with the environment is a common avoidance strategy during psychedelic experiences. It should be noted, however, that all correlations of introspection and interaction with AVE subscales were relatively weak. Therefore, these results should be considered preliminary and interpreted carefully. That only weak correlations with AVE were found is arguably so because interacting with the environment can have innumerable functions that are entirely unrelated to avoidance, especially under uncontrolled conditions. Based on this, it can be predicted that the observed weak correlations will be more pronounced in controlled contexts where introspection is more strongly encouraged (e.g. dosing sessions following the current standard protocol in psychedelic therapy studies; Garcia-Romeu and Richards, 2018). In contrast, and unsurprisingly, the introspection scale was strongly correlated with ACE, confirming the view that introspection is indeed necessary for acceptance-promoting learning processes to occur in psychedelic states. In summary, our results provide preliminary evidence that introspective states and interaction with the environment are two complementary aspects of the psychedelic experience that are functionally related to acceptance- and avoidance-promoting learning processes. More research into their relationship with ACE and AVE subaspects in the context of psychedelic therapies could help inform the refinement of therapeutic strategies for mitigating overextensive challenging experiences via sensory stimulation and motor activity (e.g. passive visual stimulation, basic visuomotor tasks, or movement and body work). Related to this, and well compatible with our results, a recent neuroimaging study (Mediano et al., 2020) found that visual stimulation under LSD not only reduced brain entropy but also weakened the correlation between entropy and psychometric measures of acute subjective drug effects. Similar studies may soon be conducted in clinical populations and psychedelic therapy settings, and this could help strengthen the evidence base for how to optimally harness context factors in psychedelic therapy. To maximize the potential knowledge to be gained from such studies, the use of psychometric tools specifically designed to capture the psychotherapeutic processes that can occur in psychedelic states, such as the APEQ, should be considered.

The present results may suggest that the current standard protocol for psychedelic therapy with its emphasis on introspection and limited interaction creates an optimal context for acceptance-promoting psychedelic experiences to occur. Relatedly, previous studies have provided evidence that verbal interactions during psilocybin sessions may hinder the emergence of mystical-type experiences (Russ et al., 2019a,b). It is noteworthy, however, that there are other potential mediators of therapeutic effects for which a more interactive setting is likely conducive, such as experiences of social connectedness (Kettner et al., 2021) or nature-relatedness (Gandy et al., 2020). Accordingly, it may be advisable that introspection and interaction be varied strategically depending on whether the targeted psychotherapeutic process is focused on problems or rather on resources (see Grawe, 1997).

### Methodological implications

The APEQ and its short form, the APEQ-S, are to our knowledge the first research instruments that have been purposefully designed to capture complementary aspects of psychedelic experiences. One argument in favor of using such complementarity-based rather than one-sided instruments whenever possible is related to the circumstance that psychedelics can increase suggestibility (Carhart-Harris et al., 2015). Against this backdrop, it should be considered that the use of one-sided questionnaires may run the risk of creating “psychometric echo chambers” where test subjects are primed with certain ideas about what they should have experienced. Likewise, one-sided instruments may lead researchers investigating potential mechanisms of psychedelic-induced change to selectively consider certain benefits while neglecting potential harms, or vice versa. Especially when complementary aspects of interest can be assumed to interact, as is the case with ACE and AVE, focusing on certain types of experiences without considering their interplay with complementary aspects may lead to simplistic representations of their therapeutic value and/or potential harms. In this regard, it is important to note that not only the EBI and the CEQ, but also many other self-report instruments that are used for retrospective characterization of psychedelic experiences, including measures of “oceanic boundlessness” (Studerus et al., 2010), “ego-dissolution” (Nour et al., 2016), “mystical experience” (MacLean et al., 2012), and “psychological insight” (Davis et al., 2021) use unipolar response formats (e.g. a visual analogue scale with the endpoints “no, not at all” and “extremely”). This format is suited for capturing a given aspect irrespective of potential complementary aspects which may have occurred over the course of the same experience but are missed unless they are captured by an additional complementary scale. Other questionnaires, such as a recently developed measure of “psychedelic communitas” (Kettner et al., 2021), use a bipolar response format (in this case a Likert-type scale ranging from “strongly disagree” to “strongly agree”). This format likely renders the measure sensitive to potential complementary aspects of communitas (e.g. alienation), thereby reducing one-sidedness but also potentially confounding one aspect with the other. Such confounding can indeed be intended and useful depending on the given research aims. However, in cases where researchers intend to avoid both confounding and one-sidedness when characterizing psychedelic experiences retrospectively, separate unipolar scales for each pair of complementary aspects, as exemplified by the APEQ, should be used.

#### Culturally decentered assessment of psychedelic drug effects

To our knowledge, all psychometric self-report measures that are regularly used in contemporary research for characterizing acute psychedelic experiences have been developed in one language (mostly English, a notable exception being the originally German Altered States of Consciousness Rating Scale; ASC; Dittrich, 1998; Studerus et al., 2010) and subsequently translated. One disadvantage of this successive development approach is that the original items can be culturally centered: They often contain idiosyncratic expressions specific to the original language and the associated culture, limiting the achievable equivalence of translated versions (Tanzer, 2005). To avoid this problem, here we pursued an elaborate parallel development approach where items were drafted, discussed, and revised simultaneously in English and German, including two rounds of review by a bilingual expert task force. Later in the development process, factor loadings in selection strata for both languages were used (among other criteria) to select of the final APEQ items. Subsequent replication in independent replication strata showed strong convergence between the English and the German samples, suggesting that the resulting questionnaire is indeed highly equivalent in both languages. Being decentered between English and German, the APEQ is unlikely to feature very specific idiosyncrasies of either language. We therefore anticipate that it will be relatively easy to create acceptably equivalent third-language versions of the APEQ by means of translation.

### Limitations

This study has a number of limitations. Data collection was conducted via an anonymous online survey; hence, the responses cannot be verified and undesired features such as duplicate participants cannot be ruled out. The sample is likely subject to variations in accessibility and digital competencies. Furthermore, participation bias may have led to an under-representation of individuals with negative attitudes toward psychedelics, and it is possible that highly avoidance-promoting psychedelic experiences (i.e. according to our results experiences characterized by the combination of high AVE with low ACE) were under-reported precisely because of their avoidance-promoting nature. This could indeed be reflected in the relatively low AVE scores found in this study, and may be a general problem with online surveys that are concerned with psychedelic experiences—even when they focus on challenging experiences. In a large online survey inquiring about participants’ single most psychologically difficult experience after ingesting psilocybin-containing mushrooms (Carbonaro et al., 2016), as many as 84% of participants reported having benefited from that experience. Like our present results, this may be read as evidence for the relatively low risk–benefit ratio associated with psychedelic use (Johansen and Krebs, 2015; Morgan et al., 2010; Van Amsterdam et al., 2011, 2015). However, the role of self-selection effects should also be taken into account. To include higher numbers of clearly harmful psychedelic experiences in future studies, it may be necessary to apply more specifically targeted recruitment strategies. Another important limitation of this study is the cross-sectional retrospective survey design, which is susceptible to recall biases and does not allow for causal inferences. Prospective studies are needed to further validate the proposed model, and to confirm the role of ACE and AVE as mediators between context factors and longer-term outcomes.

## Conclusion

Here, we have presented a first draft of a unified psychological model aiming to explain acceptance- and avoidance-promoting effects of psychedelic experiences. The empirical examination of this model involved the development and initial validation of a theory-based, culturally decentered self-report instrument, the APEQ, in English and German. In a bilingual online survey, the model’s components and its overarching assumptions of context-dependence, complementarity, intertwinedness, and interaction were largely confirmed. Whereas prospective studies are still needed to further validate the APEQ and refine its underlying theory, some implications related to current debates in the psychedelic field can already be drawn.

Letheby (2021) recently laid out a comprehensive argument that beneficial or therapeutic longer-term changes occasioned by psychedelic drugs are not caused by experience-independent neuropharmacological effects, nor by an (alleged) induction of non-naturalistic metaphysical ideations, but rather by a transformative re-appraisal of assumptions about the self that can be achieved by learning from the psychedelic experience. The model we propose, and the results reported here, are not only in line with this latter view, but also extend it to less beneficial or harmful effects. Psychedelic-induced psychological change can vary considerably, with outcomes evidently skewed toward the positive, but also—unfavorable conditions provided—sometimes ranging into the negative spectrum. Our model accommodates both of these possibilities by considering not only positive but also negative psychedelic-induced changes in psychological flexibility, and by specifying the underlying complementary psychological processes. Defining such processes in clear terms and providing theory-based research instruments for measuring them are necessary steps toward overcoming what has been referred to as “psychedelic exceptionalism” and establishing a demystified scientific understanding of the psychedelic state and its transformative potential (Johnson, 2021; Sanders and Zijlmans, 2021). Such an understanding is not a mere academic issue but has important implications regarding the question how psychedelic-assisted therapies should be delivered and how therapists should be trained (Gründer and Jungaberle, 2021). The model presented here, if further supported by prospective-longitudinal studies in the context of clinical trials, could be well-suited for informing answers to these questions since it builds on empirically well-established general psychotherapeutic change mechanisms that are straightforwardly related to therapeutic methods and competencies (Grawe, 1997; for a recent argument in favor of applying the “common factors” view to psychedelic therapy, see Nayak and Johnson, 2020). At the same time, the model also considers factors that are more specific to (or indeed “exceptional” for) the psychedelic state, namely, psychedelic-induced belief relaxation (Carhart-Harris and Friston, 2019) and its putative downstream effects such as increased context sensitivity (Carhart-Harris et al., 2018b) and shaping-like operant conditioning of acceptance (“learning to let go”; Wolff et al., 2020). Integrating the rapidly evolving understanding of such psychedelic-specific phenomena with existing knowledge about more general processes of psychological change (e.g. established by learning psychology and empirical psychotherapy research) is, as we hope to have clarified with the present work, a promising avenue toward making optimal use of the benefits of psychedelic drugs while also preventing potential harms.

## Supplemental Material

sj-docx-1-jop-10.1177_02698811211073758 – Supplemental material for The Acceptance/Avoidance-Promoting Experiences Questionnaire (APEQ): A theory-based approach to psychedelic drugs’ effects on psychological flexibility Click here for additional data file. Supplemental material, sj-docx-1-jop-10.1177_02698811211073758 for The Acceptance/Avoidance-Promoting Experiences Questionnaire (APEQ): A theory-based approach to psychedelic drugs’ effects on psychological flexibility by Max Wolff, Lea J Mertens, Marie Walter, Sören Enge and Ricarda Evens in Journal of Psychopharmacology

sj-docx-2-jop-10.1177_02698811211073758 – Supplemental material for The Acceptance/Avoidance-Promoting Experiences Questionnaire (APEQ): A theory-based approach to psychedelic drugs’ effects on psychological flexibility Click here for additional data file. Supplemental material, sj-docx-2-jop-10.1177_02698811211073758 for The Acceptance/Avoidance-Promoting Experiences Questionnaire (APEQ): A theory-based approach to psychedelic drugs’ effects on psychological flexibility by Max Wolff, Lea J Mertens, Marie Walter, Sören Enge and Ricarda Evens in Journal of Psychopharmacology

sj-docx-3-jop-10.1177_02698811211073758 – Supplemental material for The Acceptance/Avoidance-Promoting Experiences Questionnaire (APEQ): A theory-based approach to psychedelic drugs’ effects on psychological flexibility Click here for additional data file. Supplemental material, sj-docx-3-jop-10.1177_02698811211073758 for The Acceptance/Avoidance-Promoting Experiences Questionnaire (APEQ): A theory-based approach to psychedelic drugs’ effects on psychological flexibility by Max Wolff, Lea J Mertens, Marie Walter, Sören Enge and Ricarda Evens in Journal of Psychopharmacology

sj-docx-4-jop-10.1177_02698811211073758 – Supplemental material for The Acceptance/Avoidance-Promoting Experiences Questionnaire (APEQ): A theory-based approach to psychedelic drugs’ effects on psychological flexibility Click here for additional data file. Supplemental material, sj-docx-4-jop-10.1177_02698811211073758 for The Acceptance/Avoidance-Promoting Experiences Questionnaire (APEQ): A theory-based approach to psychedelic drugs’ effects on psychological flexibility by Max Wolff, Lea J Mertens, Marie Walter, Sören Enge and Ricarda Evens in Journal of Psychopharmacology

sj-docx-5-jop-10.1177_02698811211073758 – Supplemental material for The Acceptance/Avoidance-Promoting Experiences Questionnaire (APEQ): A theory-based approach to psychedelic drugs’ effects on psychological flexibility Click here for additional data file. Supplemental material, sj-docx-5-jop-10.1177_02698811211073758 for The Acceptance/Avoidance-Promoting Experiences Questionnaire (APEQ): A theory-based approach to psychedelic drugs’ effects on psychological flexibility by Max Wolff, Lea J Mertens, Marie Walter, Sören Enge and Ricarda Evens in Journal of Psychopharmacology

## Notes

1. Shaping (Skinner, 1953) is a conditioning procedure where differential reinforcement of successive approximations is used to gradually change a subject’s spontaneous behavior towards a target behavior.

2. The use of the term *complementarity* in the present work is inspired by (but not actually related to) the complementarity principle in quantum mechanics, that is, the circumstance that objects can have certain pairs of complementary properties which cannot be observed simultaneously.
