# Supplementary material for: The Acceptance/Avoidance-Promoting Experiences Questionnaire (APEQ): A theory-based approach to psychedelic drugs’ effects on psychological flexibility
Source: J Psychopharmacol. 2022 Mar 7;36(3):387–408. doi: 10.1177/02698811211073758 (PMC8902683; doi:10.1177/02698811211073758)
Supplement: sj-docx-1-jop-10.1177_02698811211073758 – Supplemental material for The Acceptance/Avoidance-Promoting Experiences Questionnaire (APEQ): A theory-based approach to psychedelic drugs’ effects on psychological flexibility [file sj-docx-1-jop-10.1177_02698811211073758.docx]

**APEQ**

Here is a list of statements describing various thoughts, feelings, and behaviors that may have occurred during your experience. Please rate the degree to which these statements apply to your experience or parts of the experience.

To do this, please mark the appropriate position on the line below each statement **with a vertical line**.

|  | I observed my external environment. | | |
| --- | --- | --- | --- |
|  |  |  |  |
|  | **NO**, not at all |  | **YES**, extremely or absolutely |
|  |  |  |  |
|  |  |  |  |

|  | I was engaged with what was going on inside me. | | |
| --- | --- | --- | --- |
|  |  |  |  |
|  | **NO**, not at all |  | **YES**, extremely or absolutely |
|  |  |  |  |
|  |  |  |  |

|  | It seemed to me as if some kind of blockage was being resolved. | | |
| --- | --- | --- | --- |
|  |  |  |  |
|  | **NO**, not at all |  | **YES**, extremely or absolutely |
|  |  |  |  |
|  |  |  |  |

|  | I learned to better understand certain emotional states. | | |
| --- | --- | --- | --- |
|  |  |  |  |
|  | **NO**, not at all |  | **YES**, extremely or absolutely |
|  |  |  |  |
|  |  |  |  |

|  | I tried to lessen, or rid myself of, certain perceptions or bodily sensations. | | |
| --- | --- | --- | --- |
|  |  |  |  |
|  | **NO**, not at all |  | **YES**, extremely or absolutely |
|  |  |  |  |
|  |  |  |  |

|  | I learned to fear or detest certain uncomfortable feelings or sensations more strongly. | | |
| --- | --- | --- | --- |
|  |  |  |  |
|  | **NO**, not at all |  | **YES**, extremely or absolutely |
|  |  |  |  |
|  |  |  |  |

|  | I was able to accept uncomfortable thoughts or memories. | | |
| --- | --- | --- | --- |
|  |  |  |  |
|  | **NO**, not at all |  | **YES**, extremely or absolutely |
|  |  |  |  |
|  |  |  |  |

|  | I felt tormented. | | |
| --- | --- | --- | --- |
|  |  |  |  |
|  | **NO**, not at all |  | **YES**, extremely or absolutely |
|  |  |  |  |
|  |  |  |  |

|  | I had a positive emotional breakthrough. | | |
| --- | --- | --- | --- |
|  |  |  |  |
|  | **NO**, not at all |  | **YES**, extremely or absolutely |
|  |  |  |  |
|  |  |  |  |

|  | I actively engaged with my surroundings. | | |
| --- | --- | --- | --- |
|  |  |  |  |
|  | **NO**, not at all |  | **YES**, extremely or absolutely |
|  |  |  |  |
|  |  |  |  |

|  | I discovered a deeper acceptance of certain difficult feelings or sensations. | | |
| --- | --- | --- | --- |
|  |  |  |  |
|  | **NO**, not at all |  | **YES**, extremely or absolutely |
|  |  |  |  |
|  |  |  |  |

|  | I looked inside. | | |
| --- | --- | --- | --- |
|  |  |  |  |
|  | **NO**, not at all |  | **YES**, extremely or absolutely |
|  |  |  |  |
|  |  |  |  |

|  | I was open to difficult sensations or emotional states. | | |
| --- | --- | --- | --- |
|  |  |  |  |
|  | **NO**, not at all |  | **YES**, extremely or absolutely |
|  |  |  |  |
|  |  |  |  |

|  | I panicked. | | |
| --- | --- | --- | --- |
|  |  |  |  |
|  | **NO**, not at all |  | **YES**, extremely or absolutely |
|  |  |  |  |
|  |  |  |  |

|  | I tried to change my mood. | | |
| --- | --- | --- | --- |
|  |  |  |  |
|  | **NO**, not at all |  | **YES**, extremely or absolutely |
|  |  |  |  |
|  |  |  |  |

|  | I noticed that I can tolerate certain mental states less than I thought. | | |
| --- | --- | --- | --- |
|  |  |  |  |
|  | **NO**, not at all |  | **YES**, extremely or absolutely |
|  |  |  |  |
|  |  |  |  |

|  | My attention was turned inward. | | |
| --- | --- | --- | --- |
|  |  |  |  |
|  | **NO**, not at all |  | **YES**, extremely or absolutely |
|  |  |  |  |
|  |  |  |  |

|  | I looked at painful memories with openness. | | |
| --- | --- | --- | --- |
|  |  |  |  |
|  | **NO**, not at all |  | **YES**, extremely or absolutely |
|  |  |  |  |
|  |  |  |  |

|  | I learned that it is better for me not to experience certain emotional states at all. | | |
| --- | --- | --- | --- |
|  |  |  |  |
|  | **NO**, not at all |  | **YES**, extremely or absolutely |
|  |  |  |  |
|  |  |  |  |

|  | I noticed that certain thoughts or memories are not as dangerous for me as I had previously thought. | | |
| --- | --- | --- | --- |
|  |  |  |  |
|  | **NO**, not at all |  | **YES**, extremely or absolutely |
|  |  |  |  |
|  |  |  |  |

|  | I interacted with other people. | | |
| --- | --- | --- | --- |
|  |  |  |  |
|  | **NO**, not at all |  | **YES**, extremely or absolutely |
|  |  |  |  |

|  | I felt a sense of relief. | | |
| --- | --- | --- | --- |
|  |  |  |  |
|  | **NO**, not at all |  | **YES**, extremely or absolutely |
|  |  |  |  |
|  |  |  |  |

|  | I experienced a state of distress. | | |
| --- | --- | --- | --- |
|  |  |  |  |
|  | **NO**, not at all |  | **YES**, extremely or absolutely |
|  |  |  |  |
|  |  |  |  |

|  | I attempted to suppress certain emotions or thoughts. | | |
| --- | --- | --- | --- |
|  |  |  |  |
|  | **NO**, not at all |  | **YES**, extremely or absolutely |
|  |  |  |  |
|  |  |  |  |

|  | I was absorbed in my inner experience. | | |
| --- | --- | --- | --- |
|  |  |  |  |
|  | **NO**, not at all |  | **YES**, extremely or absolutely |
|  |  |  |  |
|  |  |  |  |

|  | I moved my body. | | |
| --- | --- | --- | --- |
|  |  |  |  |
|  | **NO**, not at all |  | **YES**, extremely or absolutely |
|  |  |  |  |
|  |  |  |  |

|  | Things became easier for me in a liberating way. | | |
| --- | --- | --- | --- |
|  |  |  |  |
|  | **NO**, not at all |  | **YES**, extremely or absolutely |
|  |  |  |  |
|  |  |  |  |

|  | I made efforts to avoid or control difficult feelings. | | |
| --- | --- | --- | --- |
|  |  |  |  |
|  | **NO**, not at all |  | **YES**, extremely or absolutely |
|  |  |  |  |
|  |  |  |  |

|  | I learned that certain thoughts or memories are more dangerous for me than I previously thought. | | |
| --- | --- | --- | --- |
|  |  |  |  |
|  | **NO**, not at all |  | **YES**, extremely or absolutely |
|  |  |  |  |
|  |  |  |  |

|  | I learned to appreciate certain uncomfortable feelings or sensations more. | | |
| --- | --- | --- | --- |
|  |  |  |  |
|  | **NO**, not at all |  | **YES**, extremely or absolutely |
|  |  |  |  |
|  |  |  |  |

|  | I suffered from what I was experiencing. | | |
| --- | --- | --- | --- |
|  |  |  |  |
|  | **NO**, not at all |  | **YES**, extremely or absolutely |
|  |  |  |  |
|  |  |  |  |

|  | I managed to confront a personal fear. | | |
| --- | --- | --- | --- |
|  |  |  |  |
|  | **NO**, not at all |  | **YES**, extremely or absolutely |
|  |  |  |  |
|  |  |  |  |

**APEQ Scoring Instructions**

Visual analogue scales (VAS) on APEQ questionnaires printed on DIN A4 paper (210 x 297 mm) should be precisely 100 mm long. Item scores range from 0 to 100. Each item´s score is read out by measuring the horizontal distance between the left endpoint of the VAS and the position marked by the test subject in mm. Scores on main scales, subscales, and ancillary scales are calculated as follows.

Subscales:

Accepting Response = (Item 7 + Item 13 + Item 18 +Item 32) / 4

Relief = (Item 3 + Item 9 + Item 22 + Item 27) / 4

Pro-Acceptance Insights = (Item 4 + Item 11 + Item 20 + Item 30) / 4

Avoidant Response = (Item 5 + Item 15 + Item 24 + Item 28) / 4

Distress = (Item 8 + Item 14 + Item 23 + Item 31) / 4

Pro-Avoidance Insights = (Item 6 + Item 16 + Item 19 + Item 29) / 4

Main Scales:

Acceptance-Related Experience (ACE) = (Accepting Response + Relief + Pro-Acceptance Insights) / 3

Avoidance-Related Experience (AVE) = (Avoidant Response + Distress + Pro-Avoidance Insights) / 3

Ancillary Scales:

Introspection = (Item 2 + Item 12 + Item 17 + Item 25) / 4

Interaction = (Item 1 + Item 10 + Item 21 + Item 26) / 4
