# Supplementary material for: The Acceptance/Avoidance-Promoting Experiences Questionnaire (APEQ): A theory-based approach to psychedelic drugs’ effects on psychological flexibility
Source: J Psychopharmacol. 2022 Mar 7;36(3):387–408. doi: 10.1177/02698811211073758 (PMC8902683; doi:10.1177/02698811211073758)
Supplement: sj-docx-2-jop-10.1177_02698811211073758 – Supplemental material for The Acceptance/Avoidance-Promoting Experiences Questionnaire (APEQ): A theory-based approach to psychedelic drugs’ effects on psychological flexibility [file sj-docx-2-jop-10.1177_02698811211073758.docx]

**APEQ**

Hier ist eine Liste von Aussagen, die verschiedene Gedanken, Gefühle und Verhaltensweisen beschreiben, die möglicherweise während Ihrer Erfahrung aufgetreten sind. Bitte schätzen Sie den Umfang ein, in dem diese Aussagen auf Ihre Erfahrung oder Teile der Erfahrung zutreffen.

Markieren Sie hierzu bitte auf der jeweils darunterliegenden Linie die entsprechende Stelle **mit einem senkrechten Strich**.

|  | Ich beobachtete meine äußere Umwelt. | | |
| --- | --- | --- | --- |
|  |  |  |  |
|  | **NEIN**, überhaupt nicht |  | **JA**, extrem oder absolut |
|  |  |  |  |
|  |  |  |  |

|  | Ich war mit dem beschäftigt, was in mir vorging. | | |
| --- | --- | --- | --- |
|  |  |  |  |
|  | **NEIN**, überhaupt nicht |  | **JA**, extrem oder absolut |
|  |  |  |  |
|  |  |  |  |

|  | Es kam mir vor, als würde eine Art Blockade gelöst werden. | | |
| --- | --- | --- | --- |
|  |  |  |  |
|  | **NEIN**, überhaupt nicht |  | **JA**, extrem oder absolut |
|  |  |  |  |
|  |  |  |  |

|  | Ich lernte, bestimmte Gefühlszustände besser zu verstehen. | | |
| --- | --- | --- | --- |
|  |  |  |  |
|  | **NEIN**, überhaupt nicht |  | **JA**, extrem oder absolut |
|  |  |  |  |
|  |  |  |  |

|  | Ich versuchte, bestimmte Sinneseindrücke oder Körperwahrnehmungen abzuschwächen oder loszuwerden. | | |
| --- | --- | --- | --- |
|  |  |  |  |
|  | **NEIN**, überhaupt nicht |  | **JA**, extrem oder absolut |
|  |  |  |  |
|  |  |  |  |

|  | Ich lernte, bestimmte unangenehme Gefühle oder Empfindungen stärker zu fürchten oder verabscheuen. | | |
| --- | --- | --- | --- |
|  |  |  |  |
|  | **NEIN**, überhaupt nicht |  | **JA**, extrem oder absolut |
|  |  |  |  |
|  |  |  |  |

|  | Ich konnte unangenehme Gedanken oder Erinnerungen akzeptieren. | | |
| --- | --- | --- | --- |
|  |  |  |  |
|  | **NEIN**, überhaupt nicht |  | **JA**, extrem oder absolut |
|  |  |  |  |
|  |  |  |  |

|  | Ich fühlte mich gequält. | | |
| --- | --- | --- | --- |
|  |  |  |  |
|  | **NEIN**, überhaupt nicht |  | **JA**, extrem oder absolut |
|  |  |  |  |
|  |  |  |  |

|  | Ich hatte einen positiven emotionalen Durchbruch. | | |
| --- | --- | --- | --- |
|  |  |  |  |
|  | **NEIN**, überhaupt nicht |  | **JA**, extrem oder absolut |
|  |  |  |  |
|  |  |  |  |

|  | Ich beschäftigte mich aktiv mit meiner Umgebung. | | |
| --- | --- | --- | --- |
|  |  |  |  |
|  | **NEIN**, überhaupt nicht |  | **JA**, extrem oder absolut |
|  |  |  |  |
|  |  |  |  |

|  | Ich entdeckte eine tiefere Akzeptanz von bestimmten schwierigen Gefühlen oder Empfindungen. | | |
| --- | --- | --- | --- |
|  |  |  |  |
|  | **NEIN**, überhaupt nicht |  | **JA**, extrem oder absolut |
|  |  |  |  |
|  |  |  |  |

|  | Ich schaute nach innen. | | |
| --- | --- | --- | --- |
|  |  |  |  |
|  | **NEIN**, überhaupt nicht |  | **JA**, extrem oder absolut |
|  |  |  |  |
|  |  |  |  |

|  | Ich war offen für schwierige Empfindungen oder Gefühlszustände. | | |
| --- | --- | --- | --- |
|  |  |  |  |
|  | **NEIN**, überhaupt nicht |  | **JA**, extrem oder absolut |
|  |  |  |  |
|  |  |  |  |

|  | Ich geriet in Panik. | | |
| --- | --- | --- | --- |
|  |  |  |  |
|  | **NEIN**, überhaupt nicht |  | **JA**, extrem oder absolut |
|  |  |  |  |
|  |  |  |  |

|  | Ich versuchte, meine Stimmung zu verändern. | | |
| --- | --- | --- | --- |
|  |  |  |  |
|  | **NEIN**, überhaupt nicht |  | **JA**, extrem oder absolut |
|  |  |  |  |
|  |  |  |  |

|  | Ich bemerkte, dass ich bestimmte Geisteszustände schlechter ertrage, als ich dachte. | | |
| --- | --- | --- | --- |
|  |  |  |  |
|  | **NEIN**, überhaupt nicht |  | **JA**, extrem oder absolut |
|  |  |  |  |
|  |  |  |  |

|  | Meine Aufmerksamkeit war nach innen gerichtet. | | |
| --- | --- | --- | --- |
|  |  |  |  |
|  | **NEIN**, überhaupt nicht |  | **JA**, extrem oder absolut |
|  |  |  |  |
|  |  |  |  |

|  | Ich betrachtete schmerzhafte Erinnerungen mit Offenheit. | | |
| --- | --- | --- | --- |
|  |  |  |  |
|  | **NEIN**, überhaupt nicht |  | **JA**, extrem oder absolut |
|  |  |  |  |
|  |  |  |  |

|  | Ich lernte, dass es besser für mich ist, bestimmte Gefühlszustände überhaupt nicht zu erleben. | | |
| --- | --- | --- | --- |
|  |  |  |  |
|  | **NEIN**, überhaupt nicht |  | **JA**, extrem oder absolut |
|  |  |  |  |
|  |  |  |  |

|  | Ich bemerkte, dass bestimmte Gedanken oder Erinnerungen nicht so gefährlich für mich sind, wie ich zuvor dachte. | | |
| --- | --- | --- | --- |
|  |  |  |  |
|  | **NEIN**, überhaupt nicht |  | **JA**, extrem oder absolut |
|  |  |  |  |
|  |  |  |  |

|  | Ich interagierte mit anderen Personen. | | |
| --- | --- | --- | --- |
|  |  |  |  |
|  | **NEIN**, überhaupt nicht |  | **JA**, extrem oder absolut |
|  |  |  |  |
|  |  |  |  |

|  | Ich erlebte ein Gefühl der Erleichterung. | | |
| --- | --- | --- | --- |
|  |  |  |  |
|  | **NEIN**, überhaupt nicht |  | **JA**, extrem oder absolut |
|  |  |  |  |
|  |  |  |  |

|  | Ich erlebte einen Zustand von Bedrängnis. | | |
| --- | --- | --- | --- |
|  |  |  |  |
|  | **NEIN**, überhaupt nicht |  | **JA**, extrem oder absolut |
|  |  |  |  |
|  |  |  |  |

|  | Ich versuchte, bestimmte Gefühle oder Gedanken zu unterdrücken. | | |
| --- | --- | --- | --- |
|  |  |  |  |
|  | **NEIN**, überhaupt nicht |  | **JA**, extrem oder absolut |
|  |  |  |  |
|  |  |  |  |

|  | Ich war in meine innere Erfahrung vertieft. | | |
| --- | --- | --- | --- |
|  |  |  |  |
|  | **NEIN**, überhaupt nicht |  | **JA**, extrem oder absolut |
|  |  |  |  |
|  |  |  |  |

|  | Ich bewegte meinen Körper. | | |
| --- | --- | --- | --- |
|  |  |  |  |
|  | **NEIN**, überhaupt nicht |  | **JA**, extrem oder absolut |
|  |  |  |  |
|  |  |  |  |

|  | Die Dinge wurden auf eine befreiende Art einfacher für mich. | | |
| --- | --- | --- | --- |
|  |  |  |  |
|  | **NEIN**, überhaupt nicht |  | **JA**, extrem oder absolut |
|  |  |  |  |
|  |  |  |  |

|  | Ich strengte mich an, schwierige Gefühle zu vermeiden oder zu kontrollieren. | | |
| --- | --- | --- | --- |
|  |  |  |  |
|  | **NEIN**, überhaupt nicht |  | **JA**, extrem oder absolut |
|  |  |  |  |
|  |  |  |  |

|  | Ich lernte, dass bestimmte Gedanken oder Erinnerungen gefährlicher für mich sind, als ich zuvor dachte. | | |
| --- | --- | --- | --- |
|  |  |  |  |
|  | **NEIN**, überhaupt nicht |  | **JA**, extrem oder absolut |
|  |  |  |  |
|  |  |  |  |

|  | Ich lernte, bestimmte unangenehme Gefühle oder Empfindungen mehr wertzuschätzen. | | |
| --- | --- | --- | --- |
|  |  |  |  |
|  | **NEIN**, überhaupt nicht |  | **JA**, extrem oder absolut |
|  |  |  |  |
|  |  |  |  |

|  | Ich litt unter dem, was ich erlebte. | | |
| --- | --- | --- | --- |
|  |  |  |  |
|  | **NEIN**, überhaupt nicht |  | **JA**, extrem oder absolut |
|  |  |  |  |
|  |  |  |  |

|  | Es gelang mir, mich einer persönlichen Angst zu stellen. | | |
| --- | --- | --- | --- |
|  |  |  |  |
|  | **NEIN**, überhaupt nicht |  | **JA**, extrem oder absolut |
|  |  |  |  |

**APEQ Scoring Instructions**

Visual analogue scales (VAS) on APEQ questionnaires printed on DIN A4 paper (210 x 297 mm) should be precisely 100 mm long. Item scores range from 0 to 100. Each item´s score is read out by measuring the horizontal distance between the left endpoint of the VAS and the position marked by the test subject in mm. Scores on main scales, subscales, and ancillary scales are calculated as follows.

Subscales:

Accepting Response = (Item 7 + Item 13 + Item 18 +Item 32) / 4

Relief = (Item 3 + Item 9 + Item 22 + Item 27) / 4

Pro-Acceptance Insights = (Item 4 + Item 11 + Item 20 + Item 30) / 4

Avoidant Response = (Item 5 + Item 15 + Item 24 + Item 28) / 4

Distress = (Item 8 + Item 14 + Item 23 + Item 31) / 4

Pro-Avoidance Insights = (Item 6 + Item 16 + Item 19 + Item 29) / 4

Main Scales:

Acceptance-Related Experience (ACE) = (Accepting Response + Relief + Pro-Acceptance Insights) / 3

Avoidance-Related Experience (AVE) = (Avoidant Response + Distress + Pro-Avoidance Insights) / 3

Ancillary Scales:

Introspection = (Item 2 + Item 12 + Item 17 + Item 25) / 4

Interaction = (Item 1 + Item 10 + Item 21 + Item 26) / 4
