# Supplementary material for: The Acceptance/Avoidance-Promoting Experiences Questionnaire (APEQ): A theory-based approach to psychedelic drugs’ effects on psychological flexibility
Source: J Psychopharmacol. 2022 Mar 7;36(3):387–408. doi: 10.1177/02698811211073758 (PMC8902683; doi:10.1177/02698811211073758)
Supplement: sj-docx-3-jop-10.1177_02698811211073758 – Supplemental material for The Acceptance/Avoidance-Promoting Experiences Questionnaire (APEQ): A theory-based approach to psychedelic drugs’ effects on psychological flexibility [file sj-docx-3-jop-10.1177_02698811211073758.docx]

**Supplementary Information**

The Acceptance/Avoidance-Promoting Experiences Questionnaire (APEQ): A Theory-Based Approach to Psychedelic Drugs’ Effects on Psychological Flexibility

Max Wolff^1,2,3*^, Lea J. Mertens^4^, Marie Walter^5^, Sören Enge^6^, & Ricarda Evens^2^

^1^ MIND Foundation, Berlin, Germany

^2^ Charité – Universitätsmedizin Berlin, corporate member of Freie Universität Berlin and Humboldt-Universität zu Berlin, Department of Psychiatry and Psychotherapy, Campus Charité Mitte, Berlin, Germany

^3^ Faculty of Psychology, Dresden University of Technology, Germany

^4^ Central Institute of Mental Health, Medical Faculty Mannheim, University of Heidelberg, Germany

^5^ Department of Psychology, University of Mainz, Germany

^6^ Department of Psychology, MSB Medical School Berlin, Germany

* Correspondence: Dr. Max Wolff (max.wolff@mind-foundation.org) (max.wolff@mind-foundation.org)

| Table S1. *APEQ Initial Item Pool, Descriptive Statistics, and Factor Loadings for Confirmatory Factor Analyses in the English (n=498) and German Item Selection Stratum (n=418)* | | | | | | | | | |
| --- | --- | --- | --- | --- | --- | --- | --- | --- | --- |
|  | |  | |  | Factor loadings | | | | |
|  | | Item text | |  | Before item selection | |  | After item selection | |
| Factor/  Item # | | English | German |  | English | German |  | English | German |
| Avoidant Response | | | |  |  |  |  |  |  |
|  | 3 | I tried to escape unpleasant sensations. | Ich versuchte, unangenehmen Empfindungen zu entkommen. |  | .719 | .757 |  | – | – |
|  | 7 | I sought distraction from unpleasant thoughts or memories. | Ich suchte nach Ablenkung von unangenehmen Gedanken oder Erinnerungen. |  | .612 | .647 |  | – | – |
|  | 13 | I made efforts to maintain control over my thoughts. | Ich strengte mich an, die Kontrolle über meine Gedanken zu behalten. |  | .628 | .688 |  | – | – |
|  | 14 | **I tried to lessen, or rid myself of, certain perceptions or bodily sensations.** | **Ich versuchte, bestimmte Sinneseindrücke oder Körperwahrnehmungen abzuschwächen oder loszuwerden.** |  | .652 | .690 |  | .599 | .646 |
|  | 17 | I resisted the experience. | Ich widersetzte mich der Erfahrung. |  | .679 | .766 |  | – | – |
|  | 18 | **I tried to change my mood.** | **Ich versuchte, meine Stimmung zu verändern.** |  | .654 | .756 |  | .654 | .742 |
|  | 28 | I ran away from something inside of me. | Ich lief vor etwas in meinem Inneren davon. |  | .643 | .705 |  | – | – |
|  | 46 | **I attempted to suppress certain emotions or thoughts.** | **Ich versuchte, bestimmte Gefühle oder Gedanken zu unterdrücken.** |  | .789 | .820 |  | .799 | .839 |
|  | 51 | **I made efforts to avoid or control difficult feelings.** | **Ich strengte mich an, schwierige Gefühle zu vermeiden oder zu kontrollieren.** |  | .775 | .816 |  | .799 | .839 |
| Accepting Response | | | |  |  |  |  |  |  |
|  | 5 | I faced uncomfortable facts. | Ich stellte mich unbequemen Tatsachen. |  | .588 | .659 |  | – | – |
|  | 19 | **I was able to accept uncomfortable thoughts or memories.** | **Ich konnte unangenehme Gedanken oder Erinnerungen akzeptieren.** |  | .698 | .782 |  | .685 | .777 |
|  | 25 | **I was open to difficult sensations or emotional states.** | **Ich war offen für schwierige Empfindungen oder Gefühlszustände.** |  | .761 | .835 |  | .756 | .831 |
|  | 26 | **I looked at painful memories with openness.** | **Ich betrachtete schmerzhafte Erinnerungen mit Offenheit.** |  | .795 | .811 |  | .840 | .830 |
|  | 33 | I let the experience unfold freely. | Ich ließ die Erfahrung sich frei entfalten. |  | .242 | .367 |  | – | – |
|  | 35 | I allowed painful feelings to just be there. | Ich ließ schmerzhafte Gefühle einfach da sein. |  | .629 | .704 |  | – | – |
|  | 41 | I explored unpleasant perceptions or bodily sensations with curiosity. | Ich erforschte unangenehme Sinneseindrücke oder Körperempfindungen mit Neugierde. |  | .583 | .525 |  | – | – |
|  | 47 | I accepted my mood as it was. | Ich nahm meine Stimmung so an, wie sie war. |  | .257 | .377 |  | – | – |
|  | 53 | **I managed to confront a personal fear.** | **Es gelang mir, mich einer persönlichen Angst zu stellen.** |  | .669 | .697 |  | .631 | .684 |
| Distress | | | |  |  |  |  |  |  |
|  | 8 | I was desperate. | Ich war verzweifelt. |  | .614 | .813 |  | – | – |
|  | 27 | **I felt tormented.** | **Ich fühlte mich gequält.** |  | .837 | .879 |  | .837 | .873 |
|  | 32 | I was frustrated. | Ich war frustriert. |  | .635 | .681 |  | – | – |
|  | 34 | I felt stuck in a situation with no way out. | Ich fühlte mich in einer Situation ohne Ausweg gefangen. |  | .785 | .815 |  | – | – |
|  | 39 | **I panicked.** | **Ich geriet in Panik.** |  | .836 | .796 |  | .820 | .808 |
|  | 45 | **I experienced a state of distress.** | **Ich erlebte einen Zustand von Bedrängnis.** |  | .832 | .793 |  | .838 | .800 |
|  | 55 | **I suffered from what I was experiencing.** | **Ich litt unter dem, was ich erlebte.** |  | .817 | .865 |  | .834 | .861 |
| Relief | | | |  |  |  |  |  |  |
|  | 4 | Feelings of fear dissipated. | Gefühle von Angst lösten sich auf. |  | .418 | .592 |  | – | – |
|  | 6 | **It seemed to me as if some kind of blockage was being resolved.** | **Es kam mir vor, als würde eine Art Blockade gelöst werden.** |  | .715 | .689 |  | .739 | .678 |
|  | 16 | **I had a positive emotional breakthrough.** | **Ich hatte einen positiven emotionalen Durchbruch.** |  | .777 | .731 |  | .799 | .771 |
|  | 23 | **I felt a sense of relief.** | **Ich erlebte ein Gefühl der Erleichterung.** |  | .708 | .706 |  | .689 | .714 |
|  | 31 | I had the impression that unsettling perceptions or sensations were losing their threatening nature. | Ich hatte den Eindruck, dass beunruhigende Wahrnehmungen oder Empfindungen ihre Bedrohlichkeit verloren. |  | .550 | .606 |  | – | – |
|  | 38 | I had the feeling of getting out of a stuck state. | Ich hatte das Gefühl, aus einem festgefahrenen Zustand herauszukommen. |  | .597 | .675 |  | – | – |
|  | 40 | **Things became easier for me in a liberating way.** | **Die Dinge wurden auf eine befreiende Art einfacher für mich.** |  | .800 | .827 |  | .762 | .787 |
|  | 52 | I felt how struggle turned into lightness. | Ich spürte, wie aus Anstrengung Leichtigkeit wurde. |  | .656 | .693 |  | – | – |
|  | 56 | I felt liberated. | Ich fühlte mich befreit. |  | .752 | .767 |  | – | – |
| Pro-Avoidance Insights | | | |  |  |  |  |  |  |
|  | 10 | I realized that I should control or suppress certain feelings more. | Ich stellte fest, dass ich bestimmte Gefühle stärker kontrollieren oder unterdrücken sollte. |  | .489 | .371 |  | – | – |
|  | 15 | **I learned to fear or detest certain uncomfortable feelings or sensations more strongly.** | **Ich lernte, bestimmte unangenehme Gefühle oder Empfindungen stärker zu fürchten oder verabscheuen.** |  | .612 | .507 |  | .532 | .661 |
|  | 21 | I came to understand that I should better avoid certain painful or difficult sensations. | Mir wurde klar, dass ich bestimmte schmerzhafte oder schwierige Empfindungen besser vermeiden sollte. |  | .608 | .819 |  | – | – |
|  | 36 | **I noticed that I can tolerate certain mental states less than I thought.** | **Ich bemerkte, dass ich bestimmte Geisteszustände schlechter ertrage, als ich dachte.** |  | .534 | .528 |  | .607 | .719 |
|  | 37 | **I learned that it is better for me not to experience certain emotional states at all.** | **Ich lernte, dass es besser für mich ist, bestimmte Gefühlszustände überhaupt nicht zu erleben.** |  | .642 | .529 |  | .666 | .686 |
|  | 57 | **I learned that certain thoughts or memories are more dangerous for me than I previously thought.** | **Ich lernte, dass bestimmte Gedanken oder Erinnerungen gefährlicher für mich sind, als ich zuvor dachte.** |  | .534 | .591 |  | .588 | .662 |
| Pro-Acceptance Insights | | | |  |  |  |  |  |  |
|  | 20 | I found a way to meet certain painful experiences with more warmth or compassion. | Ich fand einen Weg, bestimmten schmerzhaften Erfahrungen mit mehr Wärme oder Mitgefühl zu begegnen. |  | .790 | .803 |  | – | – |
|  | 24 | **I learned to better understand certain emotional states.** | **Ich lernte, bestimmte Gefühlszustände besser zu verstehen.** |  | .726 | .728 |  | .717 | .730 |
|  | 29 | I realized that I am better able to bear certain mental states than I thought. | Ich stellte fest, dass ich bestimmte Geisteszustände besser aushalten kann, als ich dachte. |  | .675 | .499 |  | – | – |
|  | 44 | **I discovered a deeper acceptance of certain difficult feelings or sensations.** | **Ich entdeckte eine tiefere Akzeptanz von bestimmten schwierigen Gefühlen oder Empfindungen.** |  | .834 | .849 |  | .832 | .821 |
|  | 50 | **I noticed that certain thoughts or memories are not as dangerous for me as I had previously thought.** | **Ich bemerkte, dass bestimmte Gedanken oder Erinnerungen nicht so gefährlich für mich sind, wie ich zuvor dachte.** |  | .688 | .711 |  | .682 | .715 |
|  | 54 | **I learned to appreciate certain uncomfortable feelings or sensations more.** | **Ich lernte, bestimmte unangenehme Gefühle oder Empfindungen mehr wertzuschätzen.** |  | .794 | .785 |  | .799 | .806 |
| Interaction | | | |  |  |  |  |  |  |
|  | 1 | **I observed my external environment.** | **Ich beobachtete meine äußere Umwelt.** |  | .618 | .754 |  | .637 | .741 |
|  | 9 | My attention was turned outward. | Meine Aufmerksamkeit war nach außen gerichtet. |  | .463 | .676 |  | – | – |
|  | 11 | I talked. | Ich sprach. |  | .589 | .608 |  | – | – |
|  | 22 | **I actively engaged with my surroundings.** | **Ich beschäftigte mich aktiv mit meiner Umgebung.** |  | .770 | .864 |  | .854 | .915 |
|  | 48 | **I interacted with other people.** | **Ich interagierte mit anderen Personen.** |  | .644 | .649 |  | .546 | .594 |
|  | 49 | **I moved my body.** | **Ich bewegte meinen Körper.** |  | .606 | .611 |  | .580 | .596 |
| Introspection | | | |  |  |  |  |  |  |
|  | 2 | **I was engaged with what was going on inside me.** | **Ich war mit dem beschäftigt, was in mir vorging.** |  | .614 | .715 |  | .676 | .724 |
|  | 12 | **I looked inside.** | **Ich schaute nach innen.** |  | .704 | .783 |  | .787 | .815 |
|  | 30 | **My attention was turned inward.** | **Meine Aufmerksamkeit war nach innen gerichtet.** |  | .800 | .864 |  | .852 | .902 |
|  | 42 | I was immersed within myself. | Ich war in mir selbst versunken. |  | .866 | .729 |  | – | – |
|  | 43 | **I was absorbed in my inner experience.** | **Ich war in meine innere Erfahrung vertieft.** |  | .869 | .794 |  | .748 | .713 |
| *Note.* The 32 items that were selected for further analyses and the final APEQ are written in bold font. | | | | | | | | | |

| Table S2. *Fit Indices for All Models Reported in Table S1.* | | | | | | | | | | | | | | |
| --- | --- | --- | --- | --- | --- | --- | --- | --- | --- | --- | --- | --- | --- | --- |
|  | | Model fit before item selection | | | | | |  | Model fit after item selection | | | | | |
|  | | RMSEA | | CFI | | SRMR | |  | RMSEA | | CFI | | SRMR | |
| Scale/Model | | Eng | Ger | Eng | Ger | Eng | Ger |  | Eng | Ger | Eng | Ger | Eng | Ger |
| Acceptance-Related Experience (ACE) | |  |  |  |  |  |  |  |  |  |  |  |  |  |
|  | Accepting Response | .119 | .149 | .835 | .813 | .083 | .092 |  | .048 | .020 | .995 | .999 | .015 | .008 |
|  | Relief | .086 | .068 | .929 | .959 | .038 | .035 |  | .110 | .055 | .974 | .993 | .025 | .014 |
|  | Pro-Acceptance Insights | .022 | .050 | .998 | .989 | .016 | .022 |  | .000 | .068 | 1.000 | .992 | .005 | .017 |
| Avoidance-Related Experience (AVE) | |  |  |  |  |  |  |  |  |  |  |  |  |  |
|  | Avoidant Response | .054 | .046 | .965 | .978 | .032 | .030 |  | .084 | .079 | .979 | .987 | .025 | .020 |
|  | Distress | .049 | .056 | .983 | .978 | .023 | .028 |  | .063 | .077 | .992 | .985 | .015 | .019 |
|  | Pro-Avoidance Insights | .071 | .066 | .927 | .943 | .045 | .044 |  | .018 | .102 | .998 | .954 | .016 | .026 |
| Ancillary Scales | |  |  |  |  |  |  |  |  |  |  |  |  |  |
|  | Introspection | .189 | .210 | .877 | .838 | .060 | .054 |  | .110 | .136 | .967 | .960 | .030 | .039 |
|  | Interaction | .140 | .169 | .857 | .855 | .057 | .068 |  | .148 | .031 | .953 | .998 | .028 | .010 |
| *Note.* RMSEA = Root Mean Square Error of Approximation; CFI = Comparative Fit Index; SRMR = Standardized Root Mean Residual; Eng = English sample; Ger = German sample. Note that the RMSEA is a poor indicator of fit in models with small degrees of freedom when applying conventional cutoff values (Kenny, Kaniskan, & McCoach, 2015). | | | | | | | | | | | | | | |

References:

Kenny, D. A., Kaniskan, B., & McCoach, D. B. (2015). The Performance of RMSEA in Models With Small Degrees of Freedom. *Sociological Methods & Research*, *44*(3), 486–507. https://doi.org/10.1177/0049124114543236
