# Supplementary material for: The Acceptance/Avoidance-Promoting Experiences Questionnaire (APEQ): A theory-based approach to psychedelic drugs’ effects on psychological flexibility
Source: J Psychopharmacol. 2022 Mar 7;36(3):387–408. doi: 10.1177/02698811211073758 (PMC8902683; doi:10.1177/02698811211073758)
Supplement: sj-docx-4-jop-10.1177_02698811211073758 – Supplemental material for The Acceptance/Avoidance-Promoting Experiences Questionnaire (APEQ): A theory-based approach to psychedelic drugs’ effects on psychological flexibility [file sj-docx-4-jop-10.1177_02698811211073758.docx]

**APEQ-S**

Here is a list of statements describing various thoughts, feelings, and behaviors that may have occurred during your experience. Please rate the degree to which these statements apply to your experience or parts of the experience.

To do this, please mark the appropriate position on the line below each statement **with a vertical line**.

|  | It seemed to me as if some kind of blockage was being resolved. | | |
| --- | --- | --- | --- |
|  |  |  |  |
|  | **NO**, not at all |  | **YES**, extremely or absolutely |
|  |  |  |  |
|  |  |  |  |

|  | I tried to lessen, or rid myself of, certain perceptions or bodily sensations. | | |
| --- | --- | --- | --- |
|  |  |  |  |
|  | **NO**, not at all |  | **YES**, extremely or absolutely |
|  |  |  |  |
|  |  |  |  |

|  | I learned to fear or detest certain uncomfortable feelings or sensations more strongly. | | |
| --- | --- | --- | --- |
|  |  |  |  |
|  | **NO**, not at all |  | **YES**, extremely or absolutely |
|  |  |  |  |
|  |  |  |  |

|  | I had a positive emotional breakthrough. | | |
| --- | --- | --- | --- |
|  |  |  |  |
|  | **NO**, not at all |  | **YES**, extremely or absolutely |
|  |  |  |  |
|  |  |  |  |

|  | I discovered a deeper acceptance of certain difficult feelings or sensations. | | |
| --- | --- | --- | --- |
|  |  |  |  |
|  | **NO**, not at all |  | **YES**, extremely or absolutely |
|  |  |  |  |
|  |  |  |  |

|  | I panicked. | | |
| --- | --- | --- | --- |
|  |  |  |  |
|  | **NO**, not at all |  | **YES**, extremely or absolutely |
|  |  |  |  |
|  |  |  |  |

|  | I looked at painful memories with openness. | | |
| --- | --- | --- | --- |
|  |  |  |  |
|  | **NO**, not at all |  | **YES**, extremely or absolutely |
|  |  |  |  |
|  |  |  |  |

|  | I attempted to suppress certain emotions or thoughts. | | |
| --- | --- | --- | --- |
|  |  |  |  |
|  | **NO**, not at all |  | **YES**, extremely or absolutely |
|  |  |  |  |
|  |  |  |  |

|  | I learned that certain thoughts or memories are more dangerous for me than I previously thought. | | |
| --- | --- | --- | --- |
|  |  |  |  |
|  | **NO**, not at all |  | **YES**, extremely or absolutely |
|  |  |  |  |

|  | I learned to better understand certain emotional states. | | |
| --- | --- | --- | --- |
|  |  |  |  |
|  | **NO**, not at all |  | **YES**, extremely or absolutely |
|  |  |  |  |
|  |  |  |  |

|  | I suffered from what I was experiencing. | | |
| --- | --- | --- | --- |
|  |  |  |  |
|  | **NO**, not at all |  | **YES**, extremely or absolutely |
|  |  |  |  |
|  |  |  |  |

|  | I managed to confront a personal fear. | | |
| --- | --- | --- | --- |
|  |  |  |  |
|  | **NO**, not at all |  | **YES**, extremely or absolutely |
|  |  |  |  |
|  |  |  |  |

**APEQ-S Scoring Instructions**

Visual analogue scales (VAS) on APEQ questionnaires printed on DIN A4 paper (210 x 297 mm) should be precisely 100 mm long. Item scores range from 0 to 100. Each item´s score is read out by measuring the horizontal distance between the left endpoint of the VAS and the position marked by the test subject in mm. Scores on main scales, subscales, and ancillary scales are calculated as follows:

Acceptance-Related Experience (ACE) = (Item 1 + Item 4 + Item 5 + Item 7 + Item 10 + Item 12) / 6

Avoidance-Related Experience (AVE) = (Item 2 + Item 3 + Item 6 + Item 8 + Item 9 + Item 11) / 6
