# Supplementary material for: The Acceptance/Avoidance-Promoting Experiences Questionnaire (APEQ): A theory-based approach to psychedelic drugs’ effects on psychological flexibility
Source: J Psychopharmacol. 2022 Mar 7;36(3):387–408. doi: 10.1177/02698811211073758 (PMC8902683; doi:10.1177/02698811211073758)
Supplement: sj-docx-5-jop-10.1177_02698811211073758 – Supplemental material for The Acceptance/Avoidance-Promoting Experiences Questionnaire (APEQ): A theory-based approach to psychedelic drugs’ effects on psychological flexibility [file sj-docx-5-jop-10.1177_02698811211073758.docx]

**APEQ-S**

Hier ist eine Liste von Aussagen, die verschiedene Gedanken, Gefühle und Verhaltensweisen beschreiben, die möglicherweise während Ihrer Erfahrung aufgetreten sind. Bitte schätzen Sie den Umfang ein, in dem diese Aussagen auf Ihre Erfahrung oder Teile der Erfahrung zutreffen.

Markieren Sie hierzu bitte auf der jeweils darunterliegenden Linie die entsprechende Stelle **mit einem senkrechten Strich**.

|  | Es kam mir vor, als würde eine Art Blockade gelöst werden. | | |
| --- | --- | --- | --- |
|  |  |  |  |
|  | **NEIN**, überhaupt nicht |  | **JA**, extrem oder absolut |
|  |  |  |  |
|  |  |  |  |

|  | Ich versuchte, bestimmte Sinneseindrücke oder Körperwahrnehmungen abzuschwächen oder loszuwerden. | | |
| --- | --- | --- | --- |
|  |  |  |  |
|  | **NEIN**, überhaupt nicht |  | **JA**, extrem oder absolut |
|  |  |  |  |
|  |  |  |  |

|  | Ich lernte, bestimmte unangenehme Gefühle oder Empfindungen stärker zu fürchten oder verabscheuen. | | |
| --- | --- | --- | --- |
|  |  |  |  |
|  | **NEIN**, überhaupt nicht |  | **JA**, extrem oder absolut |
|  |  |  |  |
|  |  |  |  |

|  | Ich hatte einen positiven emotionalen Durchbruch. | | |
| --- | --- | --- | --- |
|  |  |  |  |
|  | **NEIN**, überhaupt nicht |  | **JA**, extrem oder absolut |
|  |  |  |  |
|  |  |  |  |

|  | Ich entdeckte eine tiefere Akzeptanz von bestimmten schwierigen Gefühlen oder Empfindungen. | | |
| --- | --- | --- | --- |
|  |  |  |  |
|  | **NEIN**, überhaupt nicht |  | **JA**, extrem oder absolut |
|  |  |  |  |
|  |  |  |  |

|  | Ich geriet in Panik. | | |
| --- | --- | --- | --- |
|  |  |  |  |
|  | **NEIN**, überhaupt nicht |  | **JA**, extrem oder absolut |
|  |  |  |  |
|  |  |  |  |

|  | Ich betrachtete schmerzhafte Erinnerungen mit Offenheit. | | |
| --- | --- | --- | --- |
|  |  |  |  |
|  | **NEIN**, überhaupt nicht |  | **JA**, extrem oder absolut |
|  |  |  |  |
|  |  |  |  |
|  |  |  |  |

|  | Ich versuchte, bestimmte Gefühle oder Gedanken zu unterdrücken. | | |
| --- | --- | --- | --- |
|  |  |  |  |
|  | **NEIN**, überhaupt nicht |  | **JA**, extrem oder absolut |
|  |  |  |  |
|  |  |  |  |

|  | Ich lernte, dass bestimmte Gedanken oder Erinnerungen gefährlicher für mich sind, als ich zuvor dachte. | | |
| --- | --- | --- | --- |
|  |  |  |  |
|  | **NEIN**, überhaupt nicht |  | **JA**, extrem oder absolut |
|  |  |  |  |
|  |  |  |  |

|  | Ich lernte, bestimmte Gefühlszustände besser zu verstehen. | | |
| --- | --- | --- | --- |
|  |  |  |  |
|  | **NEIN**, überhaupt nicht |  | **JA**, extrem oder absolut |
|  |  |  |  |
|  |  |  |  |

|  | Ich litt unter dem, was ich erlebte. | | |
| --- | --- | --- | --- |
|  |  |  |  |
|  | **NEIN**, überhaupt nicht |  | **JA**, extrem oder absolut |
|  |  |  |  |
|  |  |  |  |

|  | Es gelang mir, mich einer persönlichen Angst zu stellen. | | |
| --- | --- | --- | --- |
|  |  |  |  |
|  | **NEIN**, überhaupt nicht |  | **JA**, extrem oder absolut |
|  |  |  |  |

**APEQ-S Scoring Instructions**

Visual analogue scales (VAS) on APEQ questionnaires printed on DIN A4 paper (210 x 297 mm) should be precisely 100 mm long. Item scores range from 0 to 100. Each item´s score is read out by measuring the horizontal distance between the left endpoint of the VAS and the position marked by the test subject in mm. Scores on main scales, subscales, and ancillary scales are calculated as follows:

Acceptance-Related Experience (ACE) = (Item 1 + Item 4 + Item 5 + Item 7 + Item 10 + Item 12) / 6

Avoidance-Related Experience (AVE) = (Item 2 + Item 3 + Item 6 + Item 8 + Item 9 + Item 11) / 6
